# Mass Cytometry as a Tool for Investigating Senescence in Multiple Model Systems

**DOI:** 10.3390/cells12162045

**Published:** 2023-08-11

**Authors:** Amina Abdul-Aziz, Raymond D. Devine, Justin M. Lyberger, Hsiaochi Chang, Amy Kovacs, James R. Lerma, Andrew M. Rogers, John C. Byrd, Erin Hertlein, Gregory K. Behbehani

**Affiliations:** 1 Department of Internal Medicine, University of Cincinnati, Cincinnati, OH 45221, USA; abdulaan@ucmail.uc.edu (A.A.-A.);; 2 Department of Medicine, Division of Hematology, The Ohio State University Comprehensive Cancer Center, The Ohio State University, Columbus, OH 43210, USA; 3 Maine Medical Center, Portland, ME 04102, USA; 4 Tufts University School of Medicine, Boston, MA 02111, USA; 5 Pelotonia Institute for Immuno-Oncology, The James Comprehensive Cancer Center, The Ohio State University, Columbus, OH 43210, USA

**Keywords:** immunology, mass cytometry, aging, senescence, cell cycle

## Abstract

Cellular senescence is a durable cell cycle arrest as a result of the finite proliferative capacity of cells. Senescence responds to both intrinsic and extrinsic cellular stresses, such as aging, mitochondrial dysfunction, irradiation, and chemotherapy. Here, we report on the use of mass cytometry (MC) to analyze multiple model systems and demonstrate MC as a platform for senescence analysis at the single-cell level. We demonstrate changes to p16 expression, cell cycling fraction, and histone tail modifications in several established senescent model systems and using isolated human T cells. In bone marrow mesenchymal stromal cells (BMSCs), we show increased p16 expression with subsequent passage as well as a reduction in cycling cells and open chromatin marks. In WI-38 cells, we demonstrate increased p16 expression with both culture-induced senescence and oxidative stress-induced senescence (OSIS). We also use Wanderlust, a trajectory analysis tool, to demonstrate how p16 expression changes with histone tail modifications and cell cycle proteins. Finally, we demonstrate that repetitive stimulation of human T cells with CD3/CD28 beads induces an exhausted phenotype with increased p16 expression. This p16-expressing population exhibited higher expression of exhaustion markers such as EOMES and TOX. This work demonstrates that MC is a useful platform for studying senescence at a single-cell protein level, and is capable of measuring multiple markers of senescence at once with high confidence, thereby improving our understanding of senescent pathways.

## 1. Introduction

Cellular senescence was originally defined by Hayflick and Moore as a durable cell cycle arrest as a result of the finite proliferative capacity of cultured human fibroblasts and originally coined replicative senescence [1,2]. It is now understood that senescence can be a response to a number of intrinsic and extrinsic cellular stresses including oxidative stress, mitochondrial dysfunction, irradiation, chemotherapy, and tumor promoting gene activation [3,4,5,6,7]. Since first described in 1961, the multiple mechanisms of cellular senescence have been elucidated and understood to involve multiple contributing pathways. The primary driver and indicator of senescence is activation of p21 (CIP1/WAF) and p16 (INK4A) pathways [8,9,10,11]. Both p21 and p16 function as inhibitors of cyclin dependent kinases preventing the phosphorylation of retinoblastoma protein (pRb) thereby suppressing cellular proliferation leading to senescence. The upregulation of p21 occurs before p16 and is responsible for determining cellular fate and entry into senescence. Once a cell has committed to senescence, the expression of p16 increases and the expression of phosphorylated retinoblastoma protein decreases, resulting in division arrest. More recently, changes in chromatin structure, particularly within growth-related genes, have been described by several groups [12,13,14,15,16]. These changes are affected through modifications to the histones, integral modifiers of DNA structure, which change chromatin accessibility. A reduction in open histone modifiers such as H3K4me3 and H3K9ac are noted on these DNA regions while markers of gene repression or chromatin closure such as H3K27me3 increase [17,18,19]. There is an increase in these hypermethylated regions in genes that are responsible for cellular growth as the cell enters senescence and loses the ability to divide again. Senescence as a consequence of aging has been the focus of several research groups and p16 is the gold standard marker of in vivo senescence [8,20]. While p16 increases in aging, the expression of p16 is typically asynchronous in nature. Thus, in any culture, there is frequently a subset of cells that enter senescence from increased p16 expression while neighboring cells will not necessarily express markers of senescence [21,22]. The expression of p16 can be quite specific within a given tissue with some studies showing increased p16 expression in the proximal segment of the aorta compared to the distal segment [23,24]. This specific expression and regulation of p16 warrants special consideration in the immune system, which comprises a multitude of cell types and functions. In the complex heterogeneity of the immune system, T cells in particular can show increase in the expression of senescence markers that correspond to increased age, and T cell senescence has been shown to play an immunosuppressive role [11,21,25,26,27,28,29,30]. The mechanisms of T cell senescence are still incompletely understood but represent an important aspect of senescence that can be applied clinically. For instance, T cell senescence can negatively impact immunotherapies such as the use of chimeric antigen receptor (CAR) T cells or immune checkpoint inhibitors [31,32]. T cell receptor (TCR) signaling is needed for CAR T cell efficacy and this can be compromised in senescent T cells [33]. Furthermore, as individuals age, the TCR diversity can decline, especially in CD8+ T cells as a consequence of senescence [34]. Syngeneic murine studies of exhausted/senescent CD8+ T cells demonstrated that the exhausted T cells negatively affected the ability of the CAR T cells to expand effectively and reduced leukemic cell clearance [35]. The detection and understanding of senescence markers then are important in both basic and clinical science. There is thus tremendous utility to the development of better biomarkers of cellular senescence.

While numerous in vitro models have been developed to understand pathways and biomarkers involved in senescence, only the original WI-38 fibroblast cell line remains in wide use [2,36,37]. Most primary cells were isolated from cancer patients that do not respond to senescent signals and are effectively immortal, making them unsuitable for studying senescence. The WI-38 cell line, however, is a primary lung fibroblast that undergoes a certain number of clonal doublings eventually resulting in terminal cell cycle arrest and senescence. The WI-38 cell line can also be treated with chemical agents such as chemotherapeutics and hydrogen peroxide (H_2_O_2_) to induce an early senescent phenotype due to DNA damage and cell cycle arrest [38,39,40]. Another model of senescence is bone marrow-derived/mesenchymal stromal cells (BMSCs). These BMSCs when cultured in vitro are capable of differentiation and a limited number of cellular divisions. BMSCs, like WI-38, will eventually reach senescence after a certain number of cellular divisions. The number of divisions is influenced by the age of the organism from which the cells were derived as senescent cells increase in relation to age [41,42,43,44,45,46]. This makes study of the BMSCs important to understand the mechanisms of senescence, especially concerning the reversal or abatement of senescence for BMSC-derived therapies. BMSCs are an ideal model to study senescence because they demonstrate several senescence characteristics that can reliably be studied in vitro. The cellular shape becomes enlarged taking on a flat appearance, SA-β-galactosidase (SA-β-gal) activity increases, DNA damage markers increase, increased heterochromatin, and p16/Rb and p53/p21 pathway activity increases. BMSCs in vivo also represent a unique cell population as cells that support homeostasis and interact directly with the immune cells [47,48]. BMSCs are potent immunoregulators and in aging have been shown to interact with immune cells and increase certain senescent markers, especially among T cells [49,50]. The ability of the immune system to adapt to challenges is necessary to respond to not only infections but also elimination of tumor cells. The inability of the immune system to respond to challenges can lead to negative outcomes such as vulnerability to viruses, vaccine resistance, and reduction in overall immune defense [51,52,53]. Defining and understanding senescence is important to understand immune cell function. Currently, there are several methods to examine or determine T cell senescence: inability of CD8+ T cells to be stimulated in vitro, reduced IL-2 production in vivo, and increased p16/p21 protein expression [25,28,29,54,55,56,57].

The study of senescence then is a complex multifaceted system relating cell cycle activity, chromatin changes, phosphorylation changes, and increases in senescence markers. A further layer or complexity is that while the increase in senescence associated with aging is considered gradual and steady, there are studies in aging model systems and humans that indicate senescence-associated aging is asynchronous and changes were nonmonotonic [58,59,60,61,62]. This is indicative that cellular senescence occurs in cellular subsets, necessitating the use of single-cell techniques especially in in vivo samples that have not been driven to senescence in in vitro conditions. Currently, most biomolecular studies of senescence utilize classical techniques such as Western blotting, immunofluorescence, etc. that do not allow for single-cell analysis. Understanding the complexity of senescence could be helped by combining these individual methods into a single single-cell platform. MC combines the aspects of flow cytometry and mass spectroscopy into a single platform [63,64,65,66]. Because of the minimal spillover of the individual mass channels, the number of parameters capable of analysis by MC is currently around 50 with a possibility of 60 parameters analyzed at once. MC has been applied to understanding immunology, cellular development, neural immune cells, cell metabolism, and cell cycle [64,65,66,67,68,69,70,71,72,73,74,75]. We describe the development of a MC-based assay for cellular senescence that enables study of surface proteins, cell cycle, intracellular proteins, and senescence-associated proteins at a single-cell level. The assay has been validated for the detection of senescence in a number of models to ensure the fidelity of senescence detection, including primary AML BMSCs, WI-38 cell replicative and H_2_O_2_-induced senescence, and finally, in vitro repeated stimulation of primary human T cells to demonstrate immune cell senescence. MC is a single-cell platform uniquely positioned to study different factors in a complex heterogeneous, asynchronous environment. The method outlined in this report enables rapid analysis of senescence at the single-cell level and with a high level of functional resolution, and should enable a variety of novel investigations into the role of senescence in aging, neoplasia, and immune cell function.

## 2. Materials and Methods

### 2.1. BMSC Culture

BMSCs were isolated from spongiform bone marrow (BM) of femur heads obtained from hip replacement surgeries performed at The Ohio State University Medical Center, or from BM aspirates collected during diagnosis of myeloid malignancies. All patients provided informed consent for the sample donation in accordance with an IRB-approved protocol and the Declaration of Helsinki. Isolated cells were grown in a 37 °C, 4% oxygen incubator in DMEM (4.5 g/L glucosE) (ThermoFisher, Waltham, MA, USA) supplemented with 10% FBS (ThermoFisher, Waltham, MA, USA) and Penicillin/Streptomycin (ThermoFisher, Waltham, MA, USA). BMSC cell purity was confirmed through phenotypic analysis using MC (Supplementary Figure S1). Cells were cultured progressively to induce senescence through replication. During selected cell passages (p), defined as early: p4, middle: p8–p10, and late passage: p14–p17, an aliquot of the cells was harvested for downstream analysis. In order to measure cell cycle state Iodo-deoxyuridine (IdU) (Sigma-Aldrich, Merck, St. Louis, MO, USA) as added to the cells at a final concentration of 10 uM for 10 min in a 37 °C incubator, fixed and stained as previously described [64]. Briefly, fixed samples were frozen until the time of mass cytometry analysis. Since the cultures are not always 100% pure BMSCs, BMSC surface markers (CD90, CD44) were used to gate on these cells and hematopoetic cell markers (CD34, CD45) were used to exclude any contaminating cells. This gating also helped remove debris, artifacts, and dead cells.

### 2.2. WI-38 Culture

WI-38 cells were obtained from the Coriell Institute for Medical Research at a low clonal population doubling level (CPDL, approximately 20). The induction of senescence in the WI38 is well established in the literature and is considered a classical system for studying oxidative stress-induced senescence. Cells were cultured in 21% oxygen using normal glucose DMEM supplemented with 10% FBS and Penicillin/Streptomycin. Cells were monitored visually and split upon reaching 75% confluency. WI-38 cells were sampled serially to study the development of the senescent phenotype as clonal population doubling level increased until the cells reached replicative senescence at approximately 60 doublings. To study oxidative stress-induced senescence (OSIS), the WI-38 cells were treated with 30 uM H_2_O_2_ in serum free DMEM for two hours before DMEM supplemented with FBS was added back. This process was repeated every 2 days for a total of 21 days. Cell aliquots harvested for downstream analysis were treated with IdU as described above. The OSIS experiment was repeated twice (*n* = 2).

### 2.3. Primary Immune Cell Culture

Normal peripheral enriched cells were obtained from Versiti from volunteers undergoing routine blood donation. All samples were irreversibly de-identified and volunteers consented in accordance with the Versiti IRB and Declaration of Helsinki. The leukopaks were processed using Ficoll and Human T Cell Enrichment cocktail according to the manufacturer. The isolated T cells were then counted and resuspended in RPMI 1640 media supplemented with FBS and Penicillin/Streptomycin before being rested for 1 day. Following this rest, T cells were resuspended in T cell growth media: RPMI-1640 supplemented with FBS, Penicillin/Streptomycin, 50 U/mL IL-2, 12 mM HEPES (ThermoFisher, Waltham, MA, USA), and Glutamax (ThermoFisher, Waltham, MA, USA). T cells were stimulated with CD3/CD28 beads at a ratio of 333,000 beads to 1 million T cells (Dynabeads, ThermoFisher, Waltham, MA, USA). T cells were stimulated with the CD3/CD28 beads for 3 days, the beads were then removed during the following 3 days of culture, and this process was repeated (3 days with stimulation and 3 days without) until the T cells demonstrated a noticeable drop in their S-phase fraction, which was tested on a cell aliquot every 3 days by utilizing the IdU procedure described above. At the time of cell harvest, a subset of the T cells was stimulated with PMA (50 ng/Ml) (Sigma-Aldrich, Merck, St. Louis, MO, USA) and Ionomycin (1 ug/mL) (STEMCELL Technologies, Vancouver, BC, Canada), and Golgi blocked with 1 x Brefeldin A (BD Biosciences, BD Bioscience, San Jose, CA, USA). The cells were stimulated and treated with Golgi blocked for 4 h before IdU incorporation and fixated with SmartTube Buffer as described previously. Unstimulated cells were processed in parallel. Isolated T cells were also cultured in the presence of MCF7 cells or with TGF-beta in order to induce senescence exhaustion. MCF7 cells were grown for approximately 1 week after thawing before coculture with T cells to ensure normal MCF7 function. The cells were split at a 1:4 ratio into a new flask and allowed to sit undisturbed in a 37 °C tissue culture incubator for 4 h to promote attachment. Attachment was confirmed visually using a bright-field microscope. Next, T cells were added at a ratio of 1:1 to the MCF7 cells and were cocultured for 48 h before removal. Following removal, the T cells were cultured in MCF7-conditioned media supplemented with Glutamax for the remainder of the experiment. CD3/CD28 activation beads were added to the T cells as described above but removed after 48 h in order to only stimulate the cells once. The T cells were harvested when IdU incorporation showed a noticeable decrease following the stimulation with PMA/Ionomycin as described above. For TGF-Beta stimulated cells, the isolated T cells were cultured in normal culture media supplemented with TGF-Beta at a concentration of 50 ng/mL for 48 h. Following TGF-Beta stimulation, the T cells were spun down and resuspended in normal T cell media. Activation beads were added as described above and removed after 48 h. The T cells were allowed to expand and were harvested when aliquots indicated a noticeable decrease in IdU incorporation and reduction in proliferation by live cell counting. Upon harvest, the T cells were stimulated with PMA/Ionomycin and incubated with IdU as described above. In all cases, the different timepoints and treatment conditions for each donor sample were always stained and analyzed on the same day using the same staining cocktail, and treatment conditions were always compared to control samples from the same donor that were stained and analyzed on the same day.

### 2.4. Mass Cytometry

Cell samples were barcoded as previously described [76]. Barcoding ensures control for any batch effect in the reported experiments. All timepoints for all samples were run together in a single barcoded plate (20 total samples; for instance, 5 BMSC preps at 4 timepoints each). This insured that all samples were stained with all markers in a single tube and run on the mass cytometer simultaneously. This typically reduces technical variation to <3% [76]. This was particularly important for the histone markers, as these antigens can be difficult to saturate with antibody. Following barcoding, cells were washed and stained for extracellular markers as described previously [67,72,77]. Cells were fixed and then permeabilized with ice cold MeOH and washed before intracellular staining as previously described [68]. Following intracellular staining, the cells were washed twice in cell staining media (CSM) before being incubated with PBS with 1:4000 dilution of 500 nM iridium intercalator pentamethylcyclopentadienyl-Ir(III)- dipyridophenazine (Fluidigm, Fluidigm, South San Francisco, CA, USA) and 1.6% PFA to stain cellular DNA and fix antibodies in place. See Supplementary Table S1 for the antibody list.

Cell events were acquired on the Helios CyTOF system (Fluidigm, South San Francisco, CA, USA) at an event rate of approximately 200–400 events/s with internally calibrated dual count detection. Noise reduction and cell extraction parameters were set as follows: cell length 8–150, convolution threshold 600, and dual count start 1. During event collection, samples were suspended in water containing 1:20 Fluidigm EQ beads, which are polystyrene beads loaded with metal lanthanides, to allow monitoring and normalization of instrument performance. These bead events were then removed according to an algorithm developed by Finck et al. [78], enabling correction of short- and long-term fluctuations in signal intensity. Following normalization, the barcoded cells were then debarcoded to analyze their samples individually [76,78,79].

### 2.5. Cell Gating and Analysis

FCS file analysis was performed using two different platforms, either Cytobank or Omiq, depending on the advanced analysis to be performed; regardless of platform, the initial gating was the same [80,81]. Initially, a singlet gate was drawn using the event length and Iridium signal to remove doublets and debris. This singlet gate was further refined by drawing a second singlet gate on Gaussian parameters that are a result of processing on the Helios (Supplementary Figure S1). Depending on the experiment and sample source, the gates were then further refined using surface markers. Additional live/dead discrimination was performed for each experiment and demonstrated in Supplementary Figure S2.

Cell cycle phases were gated according to previously described methods [70,82]. For cell cycle analysis, the S-phase cells were gated on an IdU-positive population against CyclinB1. S-phase cells form a distinct IdU-positive population that could be used to help delineate the other gates. If resolution between the G0/G1 and G2/M population was not clear from the biaxial plot, the amount of IdU incorporation was used to indirectly measure the boundary. The G0/G1 gate is then established as the remainder of the population not residing in either gate. The sum of all gates in the IdU versus CyclinB1 is 100%. The Ki67+ and pRb + populations were gated using biaxial plots of each marker versus IdU incorporation. Gates were drawn such that 90–95% of IdU+ (S-phasE) cells were within the Ki-67+ and pRb + gates. For p16 analysis, the nonsenescent sample was used to establish a gate boundary between the p16-negative and p16-positive populations.

For T cell analysis, the T cells exposed to antigen stimulation were gated to exclude cells that had not been activated due to stimulation. Living T cells were gated according to previous reports, following identifying those T cells that were living surface markers of activation and used to identify cells that showed evidence of having been activated by CD3/CD28 stimulation. Surface markers used to identify activated T cells used were CD25, HLADR, CD69, and CD39 [83,84,85,86,87,88].

For advanced analyses, Omiq was used for three different advanced analysis techniques: optSNE, UMAP, and Wanderlust. OptSNE and UMAP are both nonlinear dimension reduction tools that attempt to visualize data as interrelated clusters that differ from each other slightly in how they achieve dimensionality reduction and cluster connectivity. OptSNE is an improvement on the classical tSNE and uses divergence evaluation to reduce exaggeration and provide higher resolution in data visualization. UMAP is similar in purpose to tSNE but uses different computations to better preserve data structure for downstream visualization [89]. Finally, Wanderlust is a relatively new analysis that seeks to project cellular fate across time when starting from an early developmental process [90,91,92]. This method has been shown to demonstrate the changes in surface markers in certain immune cells and how they adjust over pseudo-time as the cell differentiates. Analysis was performed by gating initially on the p16^+^/pRb^−^ senescent population as a starting point, and running Wanderlust from the stopping time point rather than the beginning. For a full list of parameters for advanced analyses, see Supplementary Table S2.

### 2.6. Western Blotting

Cells were detached using accutase (Innovative Cell Technologies, San Diego, CA, USA), washed in ice-cold PBS, and then lysed in 1× Cell Lysis Buffer (Cell Signalling Technology, MA, USA) supplemented with proteinase and phosphatase inhibitors cocktail (Roche, Basel, Switzerland). Pierce BCA Protein Assay was used to quantify the protein lysates (ThermoFisher, Waltham, MA, USA). For immunoblotting, 15–25 μg of protein lysate was prepared in 2× or 4× Laemmeli’s sample buffer (Bio-Rad Laboratories, Hercules, CA, USA) and heated for 5 min at 100 °C. Samples were separated on gradient 4–15% sodium dodecyl sulfate polyacrylamide gel and transferred onto nitrocellulose membrane (Bio-Rad Laboratories, Hercules, CA, USA). The membranes were blocked for 1 h in Blocker BLOTTO Blocking Buffer (ThermoFisher, Waltham, MA, USA), followed by overnight incubation at 4 °C with primary antibody, followed by HRP-conjugated secondary antibodies, and visualized with chemiluminescent Pierce ECL Substrate (Thermo Fisher ScientifiC) on X-ray films.

### 2.7. Statistical Analysis and Data Presentation

Statistical analysis was performed for the cell cycle analysis for the BMSCs after sequential passaging. As the samples were collected sequentially, the earliest passage was used to normalize the cell cycle phases (G0, G1, S, G2, and M) of the later passages of the same sample. These samples (*n* = 5) were pooled together, and the data are represented as the average plus/minus the standard error of the mean. To determine statistical significance (where *p* < 0.05), each cell cycle phase was analyzed using Student’s *t*-test. For example, the pooled S-phase of the earliest passage was compared to the middle passage and the late passage separately for statistical differences. This was repeated for each cell cycle phase.

## 3. Results

### 3.1. Expression of p16 and CD26 Increases during Replicative Senescence

**Figure 1:**
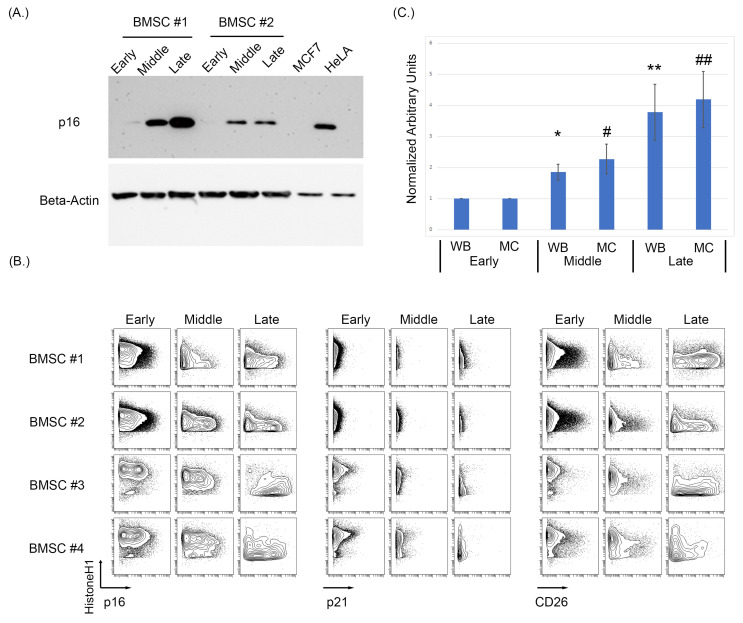
Increasing passages of BMSCs were associated with an increase in key markers of senescence. (**A**) Western blot analysis of p16 demonstrated an increase in p16 expression as passage increases in BMSCs. (**B**) Expression of p16, p21, and CD26 using MC showed an increase in p16 and CD26 expression. (**C**) Averaged normalized increase in p16 expression via Western blotting and MC between multiple cell samples from early, middle, and late BMSC passages. The average of five BMSC samples at middle and late passage was normalized to its early passage then averaged and analyzed using a *t*-test to determine significance, where *p* < 0.05 (Asterix * is used to denote significance for Western blot data, hashtag # for mass cytometry data, a single * or # denoted a *p* < 0.05, a double ** and ## denote *p* < 0.005).

As antibodies raised against p16 can cross-react to the related protein p15, we first sought to identify a p16 specific monoclonal antibody that could be used in MC studies. A number of p16 clones were tested against HeLa and MCF7cell lines as the positive and negative controls, respectively, and MCF7 overexpressing either 15 or p16 (Supplementary Figure S3). To assess the effects of replicative senescence in vitro of BMSC samples, cells were cultured and passaged with intermittent sampling until reaching senescence, defined by morphological changes and loss in proliferation (morphological changes shown in Supplementary Figure S4). Examining three distinct stages of growth representing an early (passage 4, p4), middle (p8–p10), and late passage (p14–p17), we observe a progressive increase in p16 protein level by immunoblot as BMSCs increased in passage number (Figure 1A). To assess the specificity of the antibodies used for both Western blotting and subsequent MC experiments, MCF7 and HeLa cell lines were used as negative and positive controls, respectively. As some p16 antibodies can cross-react with p15, MCF7 cells were also lipofectamine-transfected with either a p15 or p16 construct (Origene) and analyzed using both Western blot and MC (Supplementary Figure S2). The MCF7 + p16 cells showed a positive p16 population by both immunoblot and MC, while the MCF7 + p15 construct did not have a positive p16 population by either method. After p16 antibody validation, the BMSC samples were analyzed using MC to test for p16, p21, and CD26 expression levels as intracellular and surface markers of senescence (Figure 1B) [93].

MC analysis of five BMSC samples demonstrated a progressive increase in both p16 and CD26 in the early, middle, and late passages (Figure 1B). Expression of p21 only showed a slight increase in certain samples when comparing the early, middle, and late passages. The increase in median/mean p16 was statistically significant for when measured by both MC and Western blotting (WB) (*p* = 0.033 MC early compared to middle, *p* = 0.008 MC early compared to late, *p* = 0.01 WB early compared to middle, and *p* = 0.02 WB early compared to late; Figure 1C. Normalization was performed on the respective sample and then averages of the normalized values were used for the bar graphs). Senescence was validated in these samples morphologically, with senescent cells having larger body size and being visibly less dense (Supplementary Figure S4).

### 3.2. Culture-Induced Senescence of Both BMSC and WI-38 Is Associated with a Reduction in Actively Cycling Cells and an Enrichment of Cells in the G0-Phase

**Figure 2:**
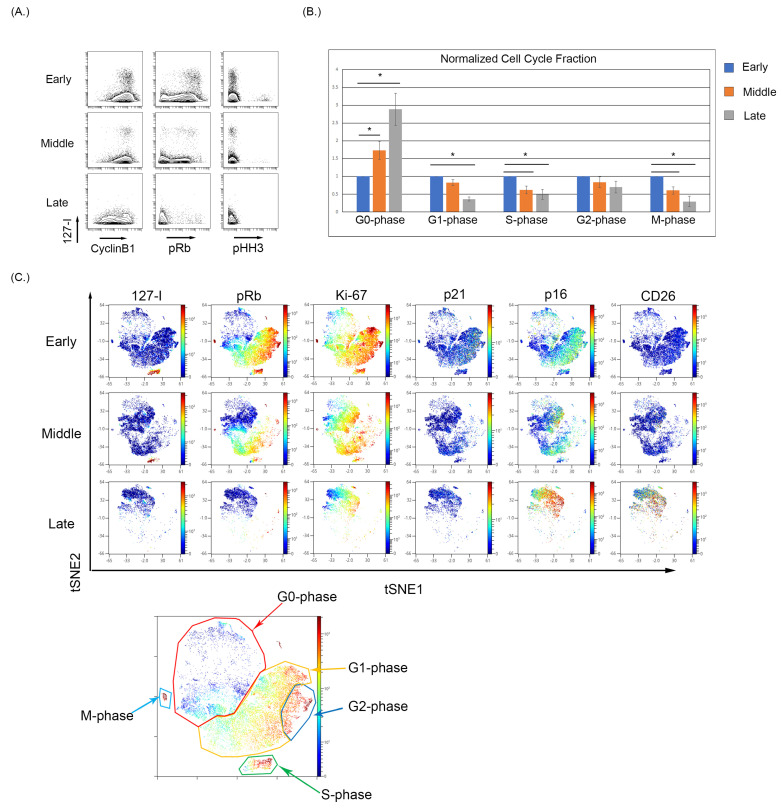
Cell cycle analysis of BMSCs with increased passage. (**A**) Reduced IdU incorporating fraction with a reduced pRb and pHH3 expression indicating a movement into a G0-phase phenotype. (**B**) The percentages of each cell cycle gate across five BMSC samples were normalized to its early passage and then averaged and analyzed using a *t*-test to determine significance where *p* < 0.05 (denoted by an Asterix *). (**C**) High dimensional analysis of BMSC samples with increasing passage demonstrate a loss in the S-phase population with a reduction in overall pRb expression and an increase in p16 expression in the G0-phase.

We observed that early passage BMSC samples show notable iododeoxyuridine (IdU) incorporation indicative of actively dividing cells in S-phase, and as the BMSC samples were continuously cultured, the fraction of IdU incorporating cells decreased (Figure 2A). The expression of pRb and pHH3 also decreases with increasing culture of BMSCs, demonstrating an increase in the G0-phase and termination of active cycling. The increase in G0-phase in cell cycle analysis was significantly increased (*p* < 0.05) between the early and late collections (Figure 2B). To visualize the relationship of senescence and cell cycle markers, optSNE was used to cluster the data based on cell cycle parameters. The optSNE demonstrated that as BMSC were progressively cultured, there was a loss of the IdU, pRb, CyclinB1, and Ki67 highly expressing cells and the majority of the cells localize to regions of the OptSNE plot composed of cells expressing markers of noncycling cells. The expression of p21 was evident in the early passage cells localizing primarily in cells in the G2-phase, and p21 expression decreased in the middle and late passages. The p16 expression was evident at low levels in early passage cells, particularly in cell in G2-phase, and can be seen localizing to the G0-phase cells in the middle and late passages, with the late passage having the highest p16 expression (Figure 2C). Finally, the expression of CD26 was absent in the early and middle passages but was increased in the late passage cells. The expression of p16 and CD26 is also noticeably overlapped in high dimensional space in the late passage cells, which demonstrated a high coexpression of p16 and CD26 (Figure 2C). Notably, the use of single-cell analysis enabled the detection of rare G0 cells expressing p16 even at early and middle passages, demonstrating that this approach can facilitate this study of senescent cells even when these cells are a small proportion of the overall sample. This also suggests that p16 expression (at least in a subset of cells) can precede many of the changes associated with senescence.

**Figure 3:**
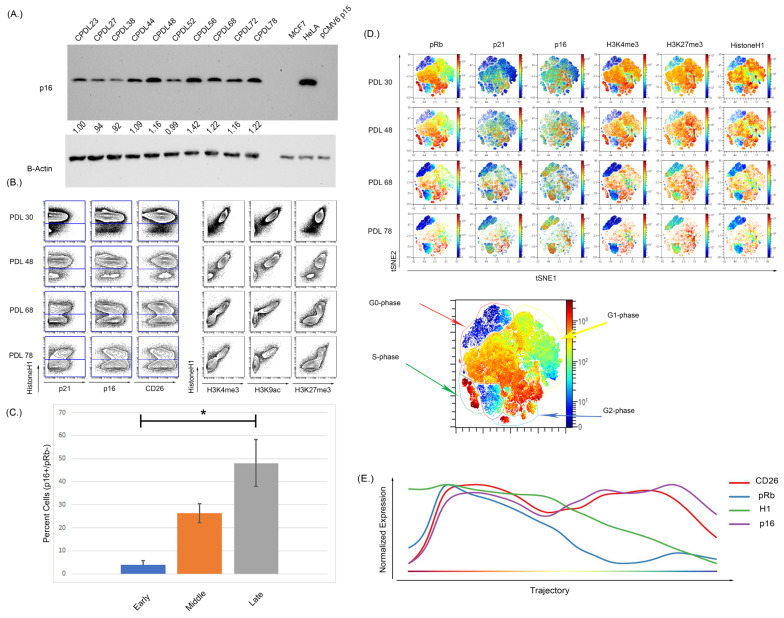
Increasing passage of WI-38 cells was associated with increased expression of senescence markers and a reduction in actively cycling cells. (**A**) Western blot analysis of WI-38 cells with increasing doubling population demonstrated an increased expression of p16. (**B**) The expression of p16 via MC shows an overall increase as doubling number increased but (**C**) more specifically, a significant expansion in a pRb low, p16 high population (*p* < 0.0001, significance denoted by an Asterix *). The analysis of histone modifications also demonstrated there was a reduction in the active transcription markers H3K4me3 and H3K9ac. H3K27me3 expression was persistently high and retained expression in later passages. (**D**) High dimensional analysis shows the distribution of p16 and histone modifications. As doubling number increases, the expression of pRb decreases alongside expression of H3K4me3. The expression of p16 increased with increasing passage number and the increased p16 expression occurred in regions of low pRb expression in the latest passage. The expression of H3K27me3 overlapped with the expression of p16 in some regions but not others, indicative of a progressive senescence phenotype. (**E**) Progressing analysis using the Wanderlust algorithm demonstrates p16 and CD26 expression increases as H1 and pRb expression decreases during the development of cellular senescence. Progression starts from G0 cells (left of plot) to senescence cells (right of plot). Analysis was performed on all cells from clonal population doubling levels (CPDLs) 30, 48, 68, and 78. Wanderlust was started from the terminal senescent population and run toward actively cycling cells.

We next confirmed these findings using WI-38 cells that underwent culture-induced senescence. The WI-38 cells were cultured from passage 23 to senescence at passage 78 and demonstrated increased p16 expression by WB and MC (Figure 3A,B). Upon repeated culture, these cells showed a noticeable reduction in the actively cycling pRb^+^ fraction with a significant expansion in the p16^+^/pRb^−^ population (Supplementary Figure S5 and Figure 3C, *p* < 0.0001 for early compared to middle, and early compared to late). High dimensional analysis of the WI- 38 cells also showed a p16^+^ population residing in the G0-phase at early passages, demonstrating an asynchronous p16-expressing population (Figure 3D). Wanderlust analysis is an algorithm that projects data as a pseudo-time graph of selected marker expression. Wanderlust analysis was performed on the WI-38, which demonstrated a progression in p16 expression that increased over the pseudo-time as senescence progresses (Figure 3E). The expression of both p16 and CD26 increased as pRb and H1 expression decreased, as the high dimensional analysis shows an overlap of pRb and H1 high expression with increasing p16 expression. To ensure that the observed changes were not due to cell death, viability was assessed by staining for cleaved PARP, GAPDH, H1, and pRb, as previously described [77]. This staining, performed in all experiments and viability gating, is shown in Supplementary Figure S2.

### 3.3. Cultured-Induced Senescence in Both BMSC and WI-38 Cells Demonstrated Senescence-Associated Changes in Global Chromatin Profile That Correlated with p16 Expression

**Figure 4:**
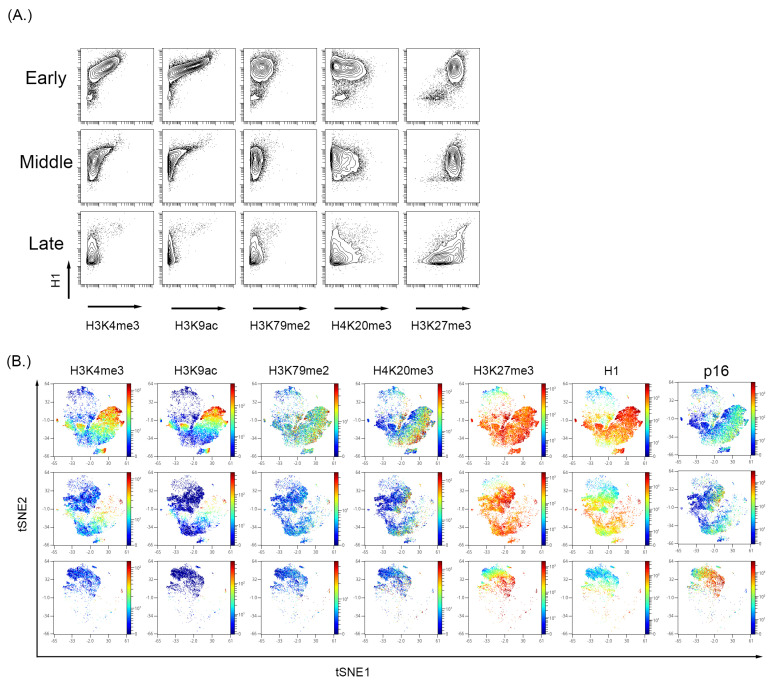
Analysis of histone modifications in the BMSC sample with increase passage. (**A**) H3K4me3 and H3K9ac decrease with a persistent expression of H3K27me3. (**B**) High dimensional analysis of the histone modifications showed that active transcription markers, H3K4me3 and H3K9ac, were reduced with increasing passage number. The expression of H3K27me3 was persistent and showed a high expression in the p16 region, indicating increased expression with increasing passage number.

To further characterize the global changes associated with senescence, BMSC and WI-38 were examined for common histone tail modifications indicative of an open or closed chromatin state. Viewing histone tail modifications utilizing MC represents a ”global” chromatin change rather than a gene or promoter-specific approach like chromatin immunoprecipitation (ChIP) [94,95]. Therefore, we also visualized p16 and cell cycle markers in high dimensional analysis to complement the analysis (Figure 3 and Figure 4). In both WI-38 and BMSC culture-induced senescence, there was a reduction in the expression level of H3K4me3 and H3K9ac alongside a reduction in total histone H1 (H1) signal (Figure 3B for WI-38 and Figure 4A for BMSC). Histone H1 has been shown previously to be depleted in certain senescence models [96]. A Western blot performed on the passages of BMSC samples showed a reduced total histone H1 signal (Supplementary Figure S6). In BMSC, H3K4me3 and H3K9ac expression showed an overall trend in reduction and were significantly reduced in the senescent cells positive for p16 and low for pRb (Figure 4 and Supplementary Figure S7) compared to all other cells. H3K27me3 can be associated with either activated or repressed transcription but remains relatively high in senescent cells [94,97,98]. In the BMSCs (Figure 4B) and WI-38 (Figure 3B and Supplementary Figure S8), the expression of H3K4me3 and H3k9ac is high initially and reduces in expression with increasing passage number. Similar to BMSCs, WI-38 cells that appeared to be senescent based on high level of p16 and low pRb, showed large decreases in expression of total histone H1, H3K4me3, and H3K9ac, as compared to all other cells. In contrast to the BMSC cells, the level of the other histone modifications (H3K9me3, and H3K27me3) was also significantly decreased, though to a lesser degree. Additionally, by the latest passage of WI-38 cells, there was minimal overlap of H3K4me3 and H3K9ac with p16 expression. H3K27me3, however, was highly expressed in the early and late passages. The signal of H3K27me3 can also be seen with higher expression in regions also expressing p16.

### 3.4. Hydrogen Peroxide Treatment of WI-38 Recapitulated the Senescent Phenotype Shown in the Culture-Induced Senescence Model

**Figure 5:**
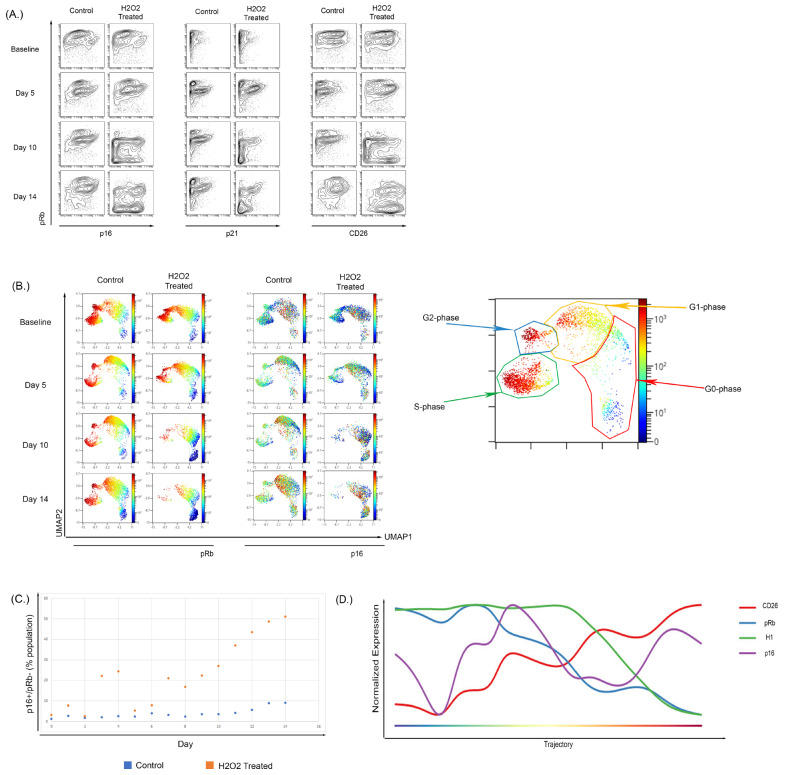
Treatment of WI-38 cells with subcytotoxic concentrations of H_2_O_2_ recapitulated a senescence phenotype. (**A**) Treatment of WI-38 cells every two days with H_2_O_2_ caused a senescent phenotype by day 10 of treatment; this phenotype showed increased overall p16 expression and the development of a low pRb-expressing population with high expression of p16. The expression of senescent surface marker CD26 showed a similar pattern with p16 expression. Overall CD26 expression increased and there was development of a pRb-low, CD26-high population. (**B**) High dimensional analysis showed an increase in p16 expression of H_2_O_2_-treated cells localizing to the G0-phase in comparison to the control samples where p16 expression was shown to be across the G2-G1-G0 phases. (**C**) Increased p16-positive, Rb-negative population in WI-38 cells untreated or treated with H_2_O_2_ over 14 days of culture. (**D**) Wanderlust analysis of the H_2_O_2_-treated WI-38 showed that there was a temporal relationship between the increase in p16 and reduction in Histone H1 and pRb.

To determine if these changes could also be observed during OSIS, WI-38 cells were treated with H_2_O_2_ [99,100,101] for 2 h every other day until the experiment ended. We observed an increase in the frequency of pRb low population that also shows an increase in CD26 expression (Figure 5A) and p16 (Figure 5A,C). The expansion in the pRb^−^/p16^+^ population could be observed in both the biaxial projections and in a high-dimensional UMAP analysis, which can provide a better snapshot of single-cell expression. In the UMAP (Figure 5B), the expression of pRb alongside p16 in the baseline and day 5 of the H_2_O_2_-treated samples is very similar to the control sample. By day 10 there was a large decrease in the S-phase population in the H_2_O_2_-treated cells (10% vs. 4%) and a reciprocal increase in the G0-phase population (17% vs. 64%), as compared to untreated control cells cultured in parallel. These effects became more pronounced by day 14 with a further decrease in S-phase cells (13% vs. 3%) and increase in the G0-phase population (26% vs. 71%). Consistent with our observations in replication-induced senescence, p16 and CD26 expression was highest in cells in the G0 and G1 phase and the overall expression of both increased as the fraction of senescent cells increased. A comparison of the fraction of cells within the senescent pRb low, p16 high population demonstrated that the fraction of cells in this population was increased in H_2_O_2_-treated cells (relative to control) at all time points, particularly days 7–14 (Figure 5C; *p* < 0.0002 by paired *t*-test).

To further clarify the progression of these changes, the results from UMAP were used to perform a Wanderlust analysis of H_2_O_2_-induced senescence in WI-38 cells. The Wanderlust analysis (Figure 5D) shows a trajectory into senescence where there is an increase in CD26 and p16 expression with a reciprocal decrease in both H1 and pRb expression. As with culture-induced senescence of BMSC and WI-38 cells, p16 could be detected in cells that still expressed markers of active cell cycling, and the progression analysis supports that this initial increase in p16 expression precedes the completely senescent state (Figure 5B). The trajectory of the Wanderlust against a biaxial cell cycle plot to demonstrate its function is shown in Supplementary Figure S9.

### 3.5. Expression of p16 and Other Markers of Senescence Can Be Observed in T Cells Exhibiting Exhaustion from Repetitive Antigen Stimulation

**Figure 6:**
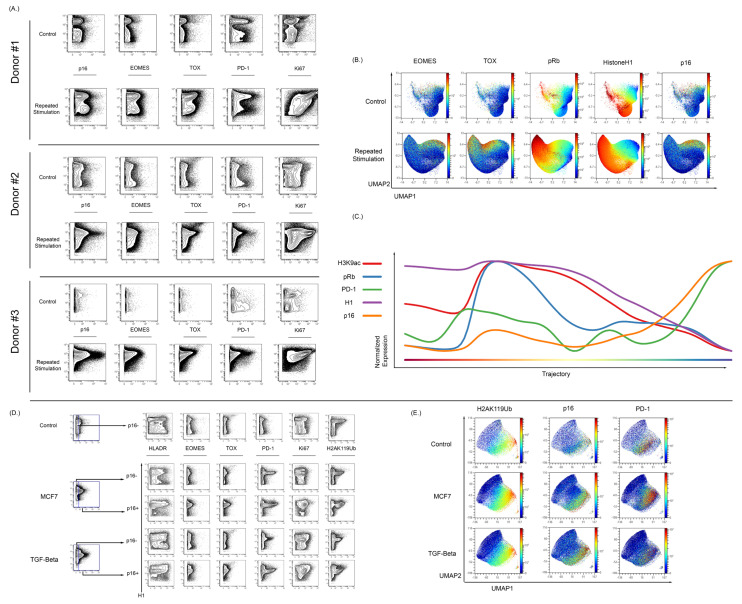
Isolated T cells were repeatedly stimulated with CD3/CD28 beads to develop a senescent/exhausted phenotype. (**A**) Repeated exposure to beads caused an increase in p16 expression when compared to control cells (*n* = 3). Cells from donor 1 were rested overnight and stimulated on days 1, 4, 7, 10, and 13, and then harvested on day 15. Donor 2 and 3 were rested for 72, stimulated on days 1, 4, 7, 10, 13, 16, 19, and 22, and harvested on day 23. The stimulated population of these stimulated cells showed increased expression of exhaustion markers EOMES, TOX, and PD-1. (**B**) High dimensional analysis of these cells showed an overlap between p16 expression and EOMES/TOX expression. The expression of EOMES and TOX also showed nonoverlapping regions of p16 expression. (**C**) Wanderlust analysis show that increased p16 expression is associated with an increase in PD-1 expression and reduction in Histone H1, pRb, and H3K9ac expression. (**D**) T cells were also cocultured in the presence of MCF7 cells and then stimulated with CD3/CD28 beads in MCF7-conditioned media for 48 hrs before bead removal. Coculture with MCF7 showed an increase in p16 expression and the p16-positive population showed increased EOMES, TOX, and PD-1. There was also an increase in the histone senescence marker, H2AK119ub, in cocultured cells when compared to control cells. T cells were also cultured with TGF-Beta (50 ng/mL) for two days to induce an exhausted and senescent phenotype. Culture with TGF-Beta was associated with increased p16 expression in comparison to control cells. These p16-positive cells showed increased expression of EOMES, TOX, and PD-1. There was also an increase in the expression of H2AK119ub with TGF-Beta-cultured T cells in vitro. (**E**) In high dimensional analysis of senescence and exhaustion markers, both the MCF7 cocultured and TGF-Beta-cultured T cells showed an overlapping expression among p16, PD-1, and H2AK119Ub.

To assess the ability of our MC approach for the study of senescence in other cell models, we stimulated normal T cells in vitro using CD3/CD28 beads. Samples were split into a control sample or a repetitive stimulation sample (2 days on 2 days off). Repetitive stimulation of T cells in vitro resulted in an increase in p16 expression when compared to matched donor controls (Figure 6A). The repetitive stimulation also resulted in an increase in the expression of EOMES, TOX, and PD-1 (Figure 6A). To understand the relationship between the exhaustion, senescence, and cycling markers, a UMAP analysis was performed alongside running the Wanderlust algorithm [90]. The UMAP demonstrated that p16 expression is mostly found in cells that appear to no longer be in the cycle. These p16^+^ cells have higher levels of exhaustion markers as compared to cells that were expressing low amounts of p16 (Figure 6B). The p16 expression increased in a fraction of T cells that continued to express some markers of active cell cycling, and progression analysis suggests that its initial expression precedes the complete loss of markers of active cell cycling. H3K9ac expression decreased in cells with increased p16 expression (Figure 6C). H3K9ac is important in T cell response to stimulation, and there is a reduced expression of H3K9ac as the T cells progress to an exhausted senescent phenotype.

To further verify if p16 levels increase during T cell exhaustion and senescence, two models of induced T exhaustion (MCF7 and TGF-beta coculture) were tested. T cells were stimulated using CD3/CD28 beads for 48 h in the presence of MCF7 cells and then allowed to expand until S-phase reduction [102,103]. Additionally, T cells were also cultured with TGF-beta before stimulation with CD3/CD28 beads for 48 h prior to expansion. T cells stimulated after exposure to either MCF7 or TGF-beta demonstrated an exhausted phenotype as compared to parallel control cells. As shown in Figure 6D, there was an increase in the p16-positive population of both the MCF7 and TGF-beta samples when compared to controls.

Relative to repetitive stimulation, the MCF7 and TGF-beta conditions lead to a smaller expression of EOMES and TOX compared to the large increase in the expression of PD-1 and fraction of PD-1 positive T-cells. This PD-1-positive population was present in both the p16-negative and p16-positive populations, but was notably increased in the p16-positive population, with the majority of p16-positive cells also expressing PD-1. 

To further confirm that these changes were associated with senescence, the histone modification H2AK119Ub was also examined in this experiment. H2AK119ub has been shown to be increased in peripheral blood of aged individuals and also increases in senescent models [95,104]. H2AK119ub was increased in both the MCF7 and TGF-beta models when compared to the control sample, and in the p16-positive population there was an increase over the p16-negative population. Visualizing these results in UMAP demonstrates overlap between p16- and H2AK119ub-expressing cells (Figure 6E). In contrast to the repetitive stimulation experiments, T cells cocultured with either MCF7 cells or TGF-beta could exhibit PD-1 and p16 expression even in the absence of markers of activation such as HLA-DR and CD39 (Supplementary Figure S10). A repeat analysis of TGF-beta treatment again demonstrated upregulation of p16 in induced senescence and again confirmed its colocalization with cells expressing markers of exhaustion (Tox, EOMES, PD-1) and the H2AK119Ub histone modification.

## 4. Discussion

### 4.1. Mass Cytometry Reliably Detects a p16^+^ Senescent Phenotype

This work demonstrates that MC can enable the study of multiple aspects of senescence at the single-cell level, thereby providing a much more detailed measurement of the development of senescence and how this interacts with other cellular processes. The use of MC for this study was to overcome two specific shortcomings in flow cytometry: (1) The multiplexing limitations of flow cytometry—MC exhibits highly parametric data, which has allowed us to study the cell cycle without staining cells for DNA content, greatly limiting the other parameters that can be measured by flow cytometry. (2) To reduce information loss due to autofluoresence—senescence in fibroblast-like cells and bone marrow mesenchymal cells, especially late passage (senescent) cells, have high autofluorescence on flow cytometry platforms (involving cell size and spectroscopic measurements) that make it very difficult to determine expression of low to medium expressed markers; additionally, autofluorescent cells are traditionally excluded from the analysis in flow cytometry, thereby risking loss of information on the more senescent cells. Although others have shown that autofluorescence can be used as a tool to detect senescent cells by flow cytometry [105], we wanted to eliminate the possibility of losing information on cells that may have low senescence and hence low autofluorescence, by opting to not rely on autofluorescence to detect senescence in our cell models. This study demonstrated that p16 expression can be measured accurately by MC and that this measurement correlated well with measures of total p16 expression by Western blot. Demonstrating the ability to evaluate p16 expression expands the abilities of MC and extends other previously established methods for MC measurement of histone modifications and cell cycle changes. The MC phenotype that we observed (p16^+^pRb^−^H1^low^CD26^+^) was consistent across multiple models of senescence and correlated with a loss in markers of open chromatin. The strengths of MC have been well established in the field of immunology, being used extensively to characterize immune cell development, differentiation, and activation [64,67,68,69,71,72,90,106,107]. This work expands the use of MC to include senescent and chromatin markers which can be measured in combination with a number of surface and functional markers to enable senescence to be studied in relation to a wide variety of other aspects of immune function, such as defining T cell exhaustion and differentiation and how it interacts with other immune cell populations.

### 4.2. Single-Cell Measurement of Senescence Reveals a Progression of p16^+^ Cell States Associated with Global Chromatin Changes

The ability to assess senescence at the single-cell level enabled us to demonstrate that p16^+^ cells demonstrated an altered global chromatin state. This chromatin state was primarily characterized by a loss of histone modifications associated with open chromatin such as H3K4me3 and H3K9ac. It is important to note that this MC approach only measures global chromatin changes. Progression analysis indicated that the loss of total histone H1 likely represents a chromatin profile associated with a fully senescent state. This conclusion is supported by the WI-38 and BMSC data where this pattern of histone markers was most prominent in cells that reached terminal senescence by all other metrics. The reduction in total H1 was confirmed by Western blot (Supplementary Figure S6) and has been previously reported [96]. This H1 depletion may be the result of ejection of chromatin into the cytoplasm (cytoplasmic chromatin fragments) [108,109,110]. It is also possible that chromatin condensation could block antibody access to the antigen binding sites; this particular assay would not be able to distinguish between these possibilities. These findings are in line with previous reports of global chromatin compaction in senescent cells [111]. The ability to measure these global chromatin changes in conjunction with other markers of the cell cycle and senescence could potentially allow for the measurement of both early and more terminal states of senescence, which can greatly enhance the study of senescence in heterogeneous biologic samples when combined with the ability to measure numerous other functional and surface markers.

### 4.3. The Expression of p16 Increases Prior to Other Markers of Terminal Senescence and Progression Analysis Suggest That p16 Expression Increases Prior to Other Senescent Cellular Markers

These experiments demonstrated that expression of p16 is dynamic and appears to increase prior to development of the classical senescent phenotype. In the UMAP and progression analyses using WI- 38 cells in both the culture and induced senescence models, p16 expression increased prior to reduced expression of pRb and H1 and prior to the maximal alterations in other chromatin markers studied (Figure 4 and Figure 5). Expression of p16 is considered the standard for the development of senescence, but p16 also performs normal cellular cycle functions [112]. The progression analysis demonstrates that p16 expression initially increases while there is still relatively high expression of H1, but as p16 expression continues to increase, the expression of H1 decreases as a final step in the progression. This decrease is preceded by a decrease in pRb. This was also evident in OSIS in WI-38 cells (Figure 5). Previous studies have demonstrated the link between senescence and ROS induction; however, this MC approach enables analysis of progressive phenotypes. This may be indicative of different types of senescent arrests, such as G2-arrest, which have been found in other studies but are hard to define [113,114]. The progression analysis demonstrated an inflection point between pRb and p16 whereas H1 expression persisted until the full development of the senescent phenotype. This was also evident in the antigen stimulation of T cells in vitro shown by the progression analysis (Figure 6C). The expression of pRb decreased before the increased expression of p16 while the chromatin markers H1 and H3K9ac remained high until p16 expression increased dramatically. While increased p16 expression is mainly found in cells that have exited the cell cycle, there are some p16-expressing cells that are still in cycle. Our high dimensional analysis indicates that some cells with high p16 expression are still in cycle, a state that appears to occur in at least a subset of cells before exiting the cell cycle. These dimensionality and progression analyses show a temporal relationship between p16 and other senescent markers that may have been underappreciated previously due to the inability to examine these global proteomic changes at a single-cell level.

### 4.4. Mass Cytometry Enables Detection of Markers of Senescence in Exhausted Primary Human T Cells

This work also demonstrates that during repetitive stimulation of T cells, p16 expression increases in a subset of T cells and the frequency of these p16^+^ cells increased over time with continued stimulation. While senescence and exhaustion are sometimes used interchangeably in the literature, they are two distinct dysfunctional states. The precision afforded by MC enabled highly parametric downstream single-cell analysis such as UMAP and Wanderlust. While each of these markers could be measured with other technologies, MC has the unique ability to examine these factors simultaneously alongside other signaling and differentiation patterns. Using highly parametric analysis methods such as UMAP, opt-SNE, etc. require high-quality data which the MC is in a unique position to generate. Using UMAP to project the expression of p16, H2AK119Ub, and PD-1 revealed an overlap between the p16 expression and H2AK119Ub and PD-1, but also shows a bright PD-1 population in the cocultured T cells that did not overlap with p16 expression. This supports a model in which a subset of exhausted cells become senescent, and the size of this fraction may have implications for the ability of immune checkpoint inhibitor therapies to restimulate the exhausted cells.

This study represents the first use of MC as a platform to study senescence using p16 protein expression as a definitive early marker of this process. Protein expression of p16 offers the opportunity to avoid measurement and quantification of p16 mRNA, which can be confounded by error and may not accurately correlate with protein levels. There are currently other aspects of MC that are being developed that can be applied to the study of senescence. Recent work by Hartman et al. has demonstrated a unique metabolic regulome in the differentiation of human cytotoxic T cells [114]. As senescent cells are still metabolically competent, but cell cycle arrested, there may be a number of changes in the metabolic regulome that can be investigated and provide new insight into the development and maintenance of senescence. The study of MC with BMSC and T cells is also important clinically. Allogeneic cells taken from a patient may not be as effective as intended due to the increased accumulation of senescent factors in the bone marrow stroma as a consequence of age and disease [41,46,115]. The measurement of senescence could be especially relevant in cellular therapies (e.g., chimeric antigen receptor T and NK cells, antiviral cytotoxic cells, or tumor-infiltrating T cells) in which understanding the proliferative qualities of the starting cells could greatly influence the effector function and ultimately the clinical efficacy of the final therapeutic product. Screening for the development of senescence in the starting cells or final product of these therapies could improve our understanding of how these treatments may fail and could facilitate the development of better methods to make these treatments more consistently effective. Since established MC assays can potentially produce results in a matter of days, this approach could have significant utility for the characterization and monitoring of cellular therapies. For hematological cancers, it could also be used to monitor both malignant and nonmalignant cells in the bone marrow or peripheral blood during and following therapy, allowing for the measurement of chemotherapy-induced senescence through the combination of senescence measurement and aberrant signaling and/or surface markers. Finally, measurement of immune cell senescence could further studies of other immunomodulatory therapies such as checkpoint inhibition for cancer therapy, immune suppression for autoimmune diseases, and monitoring of organ or stem cell transplantation.

## Data Availability

The raw data of this study are available upon request to G.K.B.

## Supplementary Materials

The following supporting information can be downloaded at: https://www.mdpi.com/article/10.3390/cells12162045/s1, Figure S1: stromal cell gating strategy; Figure S2 Gating of live cells as described previously are shown with an example of each experiment; Figure S3: Validation of a p16 antibody was done in MCF7 cells that had been transfected with either a p15 or p16 construct; Figure S4: Representative brightfield images of one of the BMSC samples; Figure S5: Changes in the p16+/pRb- expression in repeatedly cultured WI38 cells; Figure S6: Expression of p16 is highest at G1-S phase boundary and at moderate levels in G0 and G1 cells at later passages; Figure S7: Stromal cells were tested for HistoneH1 expression in order to understand chromatin accessibility; Figure S8: Changes in histone mark modifications between senescent (p16hi,pRblow; bottom histograms) vs. all other cells (top histograms) in BMSC samples from early and mid passages; Figure S9: Changes in histone mark modifications between senescent (p16hi,pRblow; bottom histograms) vs. all other cells (top histograms) in WI38 cells from early and mid passages; Figure S10: The wanderlust progression analysis used in the figures; Figure S11: The co-culture and stimulation of T cells with MCF7 cells then MCF7 conditioned media demonstrated a lack of activation following antigen stimulation; Table S1: List of antibody used; Table S2: a full list of parameters for advanced analyses.

## References

[1] Hayflick L. (1961). The establishment of a line (WISH) of human amnion cells in continuous cultivation. Exp. Cell Res..

[2] Hayflick L., Moorhead P.S. (1961). The serial cultivation of human diploid cell strains. Exp. Cell Res..

[3] Sherr C.J., DePinho R.A. (2000). Cellular senescence: Mitotic clock or culture shock?. Cell.

[4] Land H., Parada L.F., Weinberg R.A. (1983). Tumorigenic conversion of primary embryo fibroblasts requires at least two cooperating oncogenes. Nature.

[5] Kuilman T., Michaloglou C., Vredeveld L.C., Douma S., van Doorn R., Desmet C.J., Aarden L.A., Mooi W.J., Peeper D.S. (2008). Oncogene-induced senescence relayed by an interleukin-dependent inflammatory network. Cell.

[6] Moiseeva O., Bourdeau V., Roux A., Deschenes-Simard X., Ferbeyre G. (2009). Mitochondrial dysfunction contributes to oncogene-induced senescence. Mol. Cell Biol..

[7] Chang B.D., Xuan Y., Broude E.V., Zhu H., Schott B., Fang J., Roninson I.B. (1999). Role of p53 and p21waf1/cip1 in senescence-like terminal proliferation arrest induced in human tumor cells by chemotherapeutic drugs. Oncogene.

[8] Krishnamurthty J., Torrice C., Ramsey M.R., Kovalev G.I., Al-Regaiey K., Su L.S., Sharpless N.E. (2004). Ink4a/Arf expression is a biomarker of aging. J. Clin. Investig..

[9] Noren Hooten N., Evans M.K. (2017). Techniques to Induce and Quantify Cellular Senescence. J. Vis. Exp..

[10] Sharpless N.E., Sherr C.J. (2015). Forging a signature of in vivo senescence. Nat. Rev. Cancer.

[11] Vandenberk B., Brouwers B., Hatse S., Wildiers H. (2011). p16[INK4A): A central player in cellular senescence and a promising aging biomarker in elderly cancer patients. J. Geriatr. Oncol..

[12] Adams P.D. (2007). Remodeling of chromatin structure in senescent cells and its potential impact on tumor suppression and aging. Gene.

[13] Michishita E., McCord R.A., Berber E., Kioi M., Padilla-Nash H., Damian M., Cheung P., Kusumoto R., Kawahara T.L., Barrett J.C. (2008). SIRT6 is a histone H3 lysine 9 deacetylase that modulates telomeric chromatin. Nature.

[14] Bannister A.J., Kouzarides T. (2011). Regulation of chromatin by histone modifications. Cell Res..

[15] Criscione S.W., Teo Y.V., Neretti N. (2016). The Chromatin Landscape of Cellular Senescence. Trends Genet..

[16] Sun L., Yu R., Dang W. (2018). Chromatin Architectural Changes during Cellular Senescence and Aging. Genes.

[17] Garrett F.E., Emelyanov A.V., Sepulveda M.A., Flanagan P., Volpi S., Li F., Loukinov D., Eckhardt L.A., Lobanenkov V.V., Birshtein B.K. (2005). Chromatin architecture near a potential 3′ end of the igh locus involves modular regulation of histone modifications during B-Cell development and in vivo occupancy at CTCF sites. Mol. Cell Biol..

[18] Hanzelmann S., Beier F., Gusmao E.G., Koch C.M., Hummel S., Charapitsa I., Joussen S., Benes V., Brummendorf T.H., Reid G. (2015). Replicative senescence is associated with nuclear reorganization and with DNA methylation at specific transcription factor binding sites. Clin. Epigenet..

[19] Shah P.P., Donahue G., Otte G.L., Capell B.C., Nelson D.M., Cao K., Aggarwala V., Cruickshanks H.A., Rai T.S., McBryan T. (2013). Lamin B1 depletion in senescent cells triggers large-scale changes in gene expression and the chromatin landscape. Genes Dev..

[20] Zindy F., Quelle D.E., Roussel M.F., Sherr C.J. (1997). Expression of the p16INK4a tumor suppressor versus other INK4 family members during mouse development and aging. Oncogene.

[21] Dimri G.P., Lee X.H., Basile G., Acosta M., Scott C., Roskelley C., Medrano E.E., Linskens M., Rubelj I., Pereirasmith O. (1995). A Biomarker That Identifies Senescent Human-Cells in Culture and in Aging Skin in-Vivo. Proc. Natl. Acad. Sci. USA.

[22] Ressler S., Bartkova J., Niederegger H., Bartek J., Scharffetter-Kochanek K., Jansen-Durr P., Wlaschek M. (2006). p16INK4A is a robust in vivo biomarker of cellular aging in human skin. Aging Cell.

[23] Okuda K., Khan M.Y., Skurnick J., Kimura M., Aviv H., Aviv A. (2000). Telomere attrition of the human abdominal aorta: Relationships with age and atherosclerosis. Atherosclerosis.

[24] Chang E., Harley C.B. (1995). Telomere length and replicative aging in human vascular tissues. Proc. Natl. Acad. Sci. USA.

[25] Burd C.E., Peng J., Laskowski B.F., Hollyfield J.L., Zhang S., Fadda P., Yu L., Andridge R.R., Kiecolt-Glaser J.K. (2020). Association of epigenetic age and p16INK4a with markers of T cell composition in a healthy cohort. J. Gerontol. A Biol. Sci. Med. Sci..

[26] Chen L., Youssef Y., Robinson C., Ernst G.F., Carson M.Y., Young K.A., Scoville S.D., Zhang X., Harris R., Sekhri P. (2018). CD56 Expression Marks Human Group 2 Innate Lymphoid Cell Divergence from a Shared NK Cell and Group 3 Innate Lymphoid Cell Developmental Pathway. Immunity.

[27] Falandry C., Bonnefoy M., Freyer G., Gilson E. (2014). Biology of Cancer and Aging: A Complex Association With Cellular Senescence. J. Clin. Oncol..

[28] Kasakovski D., Xu L., Li Y. (2018). T cell senescence and CAR-T cell exhaustion in hematological malignancies. J. Hematol. Oncol..

[29] Liu Y., Sanoff H.K., Cho H., Burd C.E., Torrice C., Ibrahim J.G., Thomas N.E., Sharpless N.E. (2009). Expression of p16[INK4A) in peripheral blood T-cells is a biomarker of human aging. Aging Cell.

[30] Nelson J.A., Krishnamurthy J., Menezes P., Liu Y., Hudgens M.G., Sharpless N.E., Eron J.J. (2012). Expression of p16[INK4A) as a biomarker of T-cell aging in HIV-infected patients prior to and during antiretroviral therapy. Aging Cell.

[31] Cherkassky L., Morello A., Villena-Vargas J., Feng Y., Dimitrov D.S., Jones D.R., Sadelain M., Adusumilli P.S. (2016). Human CAR T cells with cell-intrinsic PD-1 checkpoint blockade resist tumor-mediated inhibition. J. Clin. Investig..

[32] Guha P., Cunetta M., Somasundar P., Espat N.J., Junghans R.P., Katz S.C. (2017). Frontline Science: Functionally impaired geriatric CAR-T cells rescued by increased alpha5beta1 integrin expression. J. Leukoc. Biol..

[33] Lanna A., Henson S.M., Escors D., Akbar A.N. (2014). The kinase p38 activated by the metabolic regulator AMPK and scaffold TAB1 drives the senescence of human T cells. Nat. Immunol..

[34] Britanova O.V., Putintseva E.V., Shugay M., Merzlyak E.M., Turchaninova M.A., Staroverov D.B., Bolotin D.A., Lukyanov S., Bogdanova E.A., Mamedov I.Z. (2014). Age-related decrease in TCR repertoire diversity measured with deep and normalized sequence profiling. J. Immunol..

[35] Yang Y., Kohler M.E., Chien C.D., Sauter C.T., Jacoby E., Yan C., Hu Y., Wanhainen K., Qin H., Fry T.J. (2017). TCR engagement negatively affects CD8 but not CD4 CAR T cell expansion and leukemic clearance. Sci. Transl. Med..

[36] Hayflick L. (1965). The Limited in Vitro Lifetime of Human Diploid Cell Strains. Exp. Cell Res..

[37] Shay J.W., Wright W.E. (2000). Hayflick, his limit, and cellular ageing. Nat. Rev. Mol. Cell Biol..

[38] Cmielova J., Havelek R., Jiroutova A., Kohlerova R., Seifrtova M., Muthna D., Vavrova J., Rezacova M. (2011). DNA damage caused by ionizing radiation in embryonic diploid fibroblasts WI-38 induces both apoptosis and senescence. Physiol. Res..

[39] Ewald J.A., Desotelle J.A., Wilding G., Jarrard D.F. (2010). Therapy-induced senescence in cancer. J. Natl. Cancer Inst..

[40] Mikula-Pietrasik J., Niklas A., Uruski P., Tykarski A., Ksiazek K. (2020). Mechanisms and significance of therapy-induced and spontaneous senescence of cancer cells. Cell Mol. Life Sci..

[41] Gnani D., Crippa S., Della Volpe L., Rossella V., Conti A., Lettera E., Rivis S., Ometti M., Fraschini G., Bernardo M.E. (2019). An early-senescence state in aged mesenchymal stromal cells contributes to hematopoietic stem and progenitor cell clonogenic impairment through the activation of a pro-inflammatory program. Aging Cell.

[42] Ksiazek K. (2009). A comprehensive review on mesenchymal stem cell growth and senescence. Rejuvenation Res..

[43] Nadeau S., Cheng A., Colmegna I., Rodier F. (2019). Quantifying Senescence-Associated Phenotypes in Primary Multipotent Mesenchymal Stromal Cell Cultures. Methods Mol. Biol..

[44] Neri S., Borzi R.M. (2020). Molecular Mechanisms Contributing to Mesenchymal Stromal Cell Aging. Biomolecules.

[45] Ren J., Stroncek D.F., Zhao Y., Jin P., Castiello L., Civini S., Wang H., Feng J., Tran K., Kuznetsov S.A. (2013). Intra-subject variability in human bone marrow stromal cell [BMSC) replicative senescence: Molecular changes associated with BMSC senescence. Stem Cell Res..

[46] Wagner W., Ho A.D., Zenke M. (2010). Different facets of aging in human mesenchymal stem cells. Tissue Eng. Part B Rev..

[47] Galderisi U., Helmbold H., Squillaro T., Alessio N., Komm N., Khadang B., Cipollaro M., Bohn W., Giordano A. (2009). In vitro senescence of rat mesenchymal stem cells is accompanied by downregulation of stemness-related and DNA damage repair genes. Stem Cells Dev..

[48] Tanabe S., Sato Y., Suzuki T., Suzuki K., Nagao T., Yamaguchi T. (2008). Gene expression profiling of human mesenchymal stem cells for identification of novel markers in early- and late-stage cell culture. J. Biochem..

[49] Mueller S.N., Germain R.N. (2009). Stromal cell contributions to the homeostasis and functionality of the immune system. Nat. Rev. Immunol..

[50] Roozendaal R., Mebius R.E. (2011). Stromal cell-immune cell interactions. Annu. Rev. Immunol..

[51] Brien J.D., Uhrlaub J.L., Hirsch A., Wiley C.A., Nikolich-Zugich J. (2009). Key role of T cell defects in age-related vulnerability to West Nile virus. J. Exp. Med..

[52] Cicin-Sain L., Smyk-Pearson S., Currier N., Byrd L., Koudelka C., Robinson T., Swarbrick G., Tackitt S., Legasse A., Fischer M. (2010). Loss of naive T cells and repertoire constriction predict poor response to vaccination in old primates. J. Immunol..

[53] Messaoudi I., Lemaoult J., Guevara-Patino J.A., Metzner B.M., Nikolich-Zugich J. (2004). Age-related CD8 T cell clonal expansions constrict CD8 T cell repertoire and have the potential to impair immune defense. J. Exp. Med..

[54] Battram A.M., Bachiller M., Martin-Antonio B. (2020). Senescence in the Development and Response to Cancer with Immunotherapy: A Double-Edged Sword. Int. J. Mol. Sci..

[55] Chou J.P., Effros R.B. (2013). T cell replicative senescence in human aging. Curr. Pharm. Des..

[56] Ferrara R., Naigeon M., Auclin E., Duchemann B., Cassard L., Jouniaux J.M., Boselli L., Grivel J., Desnoyer A., Mezquita L. (2021). Circulating T-cell Immunosenescence in Patients with Advanced Non-small Cell Lung Cancer Treated with Single-agent PD-1/PD-L1 Inhibitors or Platinum-based Chemotherapy. Clin. Cancer Res..

[57] Spaulding C., Guo W., Effros R.B. (1999). Resistance to apoptosis in human CD8+ T cells that reach replicative senescence after multiple rounds of antigen-specific proliferation. Exp. Gerontol..

[58] Herndon L.A., Schmeissner P.J., Dudaronek J.M., Brown P.A., Listner K.M., Sakano Y., Paupard M.C., Hall D.H., Driscoll M. (2002). Stochastic and genetic factors influence tissue-specific decline in ageing C. elegans. Nature.

[59] Lehallier B., Gate D., Schaum N., Nanasi T., Lee S.E., Yousef H., Moran Losada P., Berdnik D., Keller A., Verghese J. (2019). Undulating changes in human plasma proteome profiles across the lifespan. Nat. Med..

[60] Schaum N., Lehallier B., Hahn O., Palovics R., Hosseinzadeh S., Lee S.E., Sit R., Lee D.P., Losada P.M., Zardeneta M.E. (2020). Ageing hallmarks exhibit organ-specific temporal signatures. Nature.

[61] Tricoire H., Rera M. (2015). A New, Discontinuous 2 Phases of Aging Model: Lessons from Drosophila melanogaster. PLoS ONE.

[62] Marquez E.J., Chung C.H., Marches R., Rossi R.J., Nehar-Belaid D., Eroglu A., Mellert D.J., Kuchel G.A., Banchereau J., Ucar D. (2020). Sexual-dimorphism in human immune system aging. Nat. Commun..

[63] Angelo M., Bendall S.C., Finck R., Hale M.B., Hitzman C., Borowsky A.D., Levenson R.M., Lowe J.B., Liu S.D., Zhao S. (2014). Multiplexed ion beam imaging of human breast tumors. Nat. Med..

[64] Behbehani G.K., Bendall S.C., Clutter M.R., Fantl W.J., Nolan G.P. (2012). Single-cell mass cytometry adapted to measurements of the cell cycle. Cytom. A.

[65] Bjornson Z.B., Nolan G.P., Fantl W.J. (2013). Single-cell mass cytometry for analysis of immune system functional states. Curr. Opin. Immunol..

[66] Spitzer M.H., Nolan G.P. (2016). Mass Cytometry: Single Cells, Many Features. Cell.

[67] Behbehani G.K. (2019). Immunophenotyping by Mass Cytometry. Methods Mol. Biol..

[68] Behbehani G.K. (2019). Mass Cytometric Cell Cycle Analysis. Methods Mol. Biol..

[69] Behbehani G.K. (2017). Applications of Mass Cytometry in Clinical Medicine: The Promise and Perils of Clinical CyTOF. Clin. Lab. Med..

[70] Behbehani G.K. (2018). Cell Cycle Analysis by Mass Cytometry. Methods Mol. Biol..

[71] Behbehani G.K., Finck R., Samusik N., Sridhar K., Fantl W.J., Greenberg P.L., Nolan G.P. (2020). Profiling myelodysplastic syndromes by mass cytometry demonstrates abnormal progenitor cell phenotype and differentiation. Cytom. B. Clin. Cytom..

[72] Behbehani G.K., Samusik N., Bjornson Z.B., Fantl W.J., Medeiros B.C., Nolan G.P. (2015). Mass Cytometric Functional Profiling of Acute Myeloid Leukemia Defines Cell-Cycle and Immunophenotypic Properties That Correlate with Known Responses to Therapy. Cancer Discov..

[73] Di Palma S., Bodenmiller B. (2015). Unraveling cell populations in tumors by single-cell mass cytometry. Curr. Opin. Biotechnol..

[74] Fisher D.A.C., Malkova O., Engle E.K., Miner C.A., Fulbright M.C., Behbehani G.K., Collins T.B., Bandyopadhyay S., Zhou A., Nolan G.P. (2017). Mass cytometry analysis reveals hyperactive NF Kappa B signaling in myelofibrosis and secondary acute myeloid leukemia. Leukemia.

[75] Tanner S.D., Baranov V.I., Ornatsky O.I., Bandura D.R., George T.C. (2013). An introduction to mass cytometry: Fundamentals and applications. Cancer Immunol. Immunother..

[76] Zunder E.R., Finck R., Behbehani G.K., Amir El A.D., Krishnaswamy S., Gonzalez V.D., Lorang C.G., Bjornson Z., Spitzer M.H., Bodenmiller B. (2015). Palladium-based mass tag cell barcoding with a doublet-filtering scheme and single-cell deconvolution algorithm. Nat. Protoc..

[77] Devine R.D., Alkhalaileh H.S., Lyberger J.M., Behbehani G.K. (2021). Alternative methods of viability determination in single cell mass cytometry. Cytom. A.

[78] Finck R., Simonds E.F., Jager A., Krishnaswamy S., Sachs K., Fantl W., Pe’er D., Nolan G.P., Bendall S.C. (2013). Normalization of mass cytometry data with bead standards. Cytom. A.

[79] Behbehani G.K., Thom C., Zunder E.R., Finck R., Gaudilliere B., Fragiadakis G.K., Fantl W.J., Nolan G.P. (2014). Transient partial permeabilization with saponin enables cellular barcoding prior to surface marker staining. Cytom. A.

[80] Chen T.J., Kotecha N. (2014). Cytobank: Providing an analytics platform for community cytometry data analysis and collaboration. Curr. Top. Microbiol. Immunol..

[81] Belkina A.C., Ciccolella C.O., Anno R., Halpert R., Spidlen J., Snyder-Cappione J.E. (2019). Automated optimized parameters for T-distributed stochastic neighbor embedding improve visualization and analysis of large datasets. Nat. Commun..

[82] Devine R.D., Sekhri P., Behbehani G.K. (2018). Effect of storage time and temperature on cell cycle analysis by mass cytometry. Cytom. A.

[83] Lind A., Brekke K., Pettersen F.O., Mollnes T.E., Troseid M., Kvale D. (2014). A parameter for IL-10 and TGF-ss mediated regulation of HIV-1 specific T cell activation provides novel information and relates to progression markers. PLoS ONE.

[84] Wakiguchi H., Hasegawa S., Suzuki Y., Kudo K., Ichiyama T. (2015). Relationship between T-cell HLA-DR expression and intravenous immunoglobulin treatment response in Kawasaki disease. Pediatr. Res..

[85] Raczkowski F., Rissiek A., Ricklefs I., Heiss K., Schumacher V., Wundenberg K., Haag F., Koch-Nolte F., Tolosa E., Mittrucker H.W. (2018). CD39 is upregulated during activation of mouse and human T cells and attenuates the immune response to Listeria monocytogenes. PLoS ONE.

[86] Reddy M., Eirikis E., Davis C., Davis H.M., Prabhakar U. (2004). Comparative analysis of lymphocyte activation marker expression and cytokine secretion profile in stimulated human peripheral blood mononuclear cell cultures: An in vitro model to monitor cellular immune function. J. Immunol. Methods.

[87] Starska K., Glowacka E., Kulig A., Lewy-Trenda I., Brys M., Lewkowicz P. (2011). The role of tumor cells in the modification of T lymphocytes activity--the expression of the early CD69+, CD71+ and the late CD25+, CD26+, HLA/DR+ activation markers on T CD4+ and CD8+ cells in squamous cell laryngeal carcinoma. Part I. Folia Histochem. Cytobiol..

[88] Tincati C., Bellistri G.M., Ancona G., Merlini E., d’Arminio Monforte A., Marchetti G. (2012). Role of in vitro stimulation with lipopolysaccharide on T-cell activation in HIV-infected antiretroviral-treated patients. Clin. Dev. Immunol..

[89] Byerly A. (2021). Enhanced Uniform Manifold Approximation and Projection via Simultaneous Perturbation Stochastic Approximation.

[90] Bendall S.C., Davis K.L., Amir El A.D., Tadmor M.D., Simonds E.F., Chen T.J., Shenfeld D.K., Nolan G.P., Pe’er D. (2014). Single-cell trajectory detection uncovers progression and regulatory coordination in human B cell development. Cell.

[91] Becht E., McInnes L., Healy J., Dutertre C.A., Kwok I.W.H., Ng L.G., Ginhoux F., Newell E.W. (2018). Dimensionality reduction for visualizing single-cell data using UMAP. Nat. Biotechnol..

[92] McInnes L. (2020). UMAP: Uniform Manifold Approximation and Projection for Dimension Reduction. arXiv.

[93] Kim K.M., Noh J.H., Bodogai M., Martindale J.L., Yang X., Indig F.E., Basu S.K., Ohnuma K., Morimoto C., Johnson P.F. (2017). Identification of senescent cell surface targetable protein DPP4. Genes Dev..

[94] Cheung P., Vallania F., Dvorak M., Chang S.E., Schaffert S., Donato M., Rao A.M., Mao R., Utz P.J., Khatri P. (2018). Single-cell epigenetics—Chromatin modification atlas unveiled by mass cytometry. Clin. Immunol..

[95] Cheung P., Vallania F., Warsinske H.C., Donato M., Schaffert S., Chang S.E., Dvorak M., Dekker C.L., Davis M.M., Utz P.J. (2018). Single-Cell Chromatin Modification Profiling Reveals Increased Epigenetic Variations with Aging. Cell.

[96] Funayama R., Saito M., Tanobe H., Ishikawa F. (2006). Loss of linker histone H1 in cellular senescence. J. Cell Biol..

[97] Cai Y., Zhang Y., Loh Y.P., Tng J.Q., Lim M.C., Cao Z., Raju A., Lieberman Aiden E., Li S., Manikandan L. (2021). H3K27me3-rich genomic regions can function as silencers to repress gene expression via chromatin interactions. Nat. Commun..

[98] Wiles E.T., Selker E.U. (2017). H3K27 methylation: A promiscuous repressive chromatin mark. Curr. Opin. Genet. Dev..

[99] Lee A.C., Fenster B.E., Ito H., Takeda K., Bae N.S., Hirai T., Yu Z.X., Ferrans V.J., Howard B.H., Finkel T. (1999). Ras proteins induce senescence by altering the intracellular levels of reactive oxygen species. J. Biol. Chem..

[100] Pu X., Yu S., Fan W., Liu L., Ma X., Ren J. (2015). Guiqi polysaccharide protects the normal human fetal lung fibroblast WI-38 cells from H_2_O_2_-induced premature senescence. Int. J. Clin. Exp. Pathol..

[101] Zdanov S., Remacle J., Toussaint O. (2006). Establishment of H_2_O_2_-induced premature senescence in human fibroblasts concomitant with increased cellular production of H_2_O_2_. Ann. N. Y. Acad. Sci..

[102] Saleh R., Toor S.M., Khalaf S., Elkord E. (2019). Breast Cancer Cells and PD-1/PD-L1 Blockade Upregulate the Expression of PD-1, CTLA-4, TIM-3 and LAG-3 Immune Checkpoints in CD4^+^ T Cells. Vaccines.

[103] Yang Z.Z., Grote D.M., Xiu B., Ziesmer S.C., Price-Troska T.L., Hodge L.S., Yates D.M., Novak A.J., Ansell S.M. (2014). TGF-beta upregulates CD70 expression and induces exhaustion of effector memory T cells in B-cell non-Hodgkin’s lymphoma. Leukemia.

[104] Arenzana T.L., Lianoglou S., Seki A., Eidenschenk C., Cheung T., Seshasayee D., Hagenbeek T., Sambandam A., Noubade R., Peng I. (2018). Tumor suppressor BAP1 is essential for thymic development and proliferative responses of T lymphocytes. Sci. Immunol..

[105] Malavolta M., Giacconi R., Piacenza F., Strizzi S., Cardelli M., Bigossi G., Provinciali M. (2022). Simple Detection of Unstained Live Senescent Cells with Imaging Flow Cytometry. Cells.

[106] Bagwell C.B., Hunsberger B., Hill B., Herbert D., Bray C., Selvanantham T., Li S., Villasboas J.C., Pavelko K., Strausbauch M. (2020). Multi-site reproducibility of a human immunophenotyping assay in whole blood and peripheral blood mononuclear cells preparations using CyTOF technology coupled with Maxpar Pathsetter, an automated data analysis system. Cytom. B. Clin. Cytom..

[107] Bendall S.C., Simonds E.F., Qiu P., Amir El A.D., Krutzik P.O., Finck R., Bruggner R.V., Melamed R., Trejo A., Ornatsky O.I. (2011). Single-cell mass cytometry of differential immune and drug responses across a human hematopoietic continuum. Science.

[108] Dou Z., Xu C., Donahue G., Shimi T., Pan J.A., Zhu J., Ivanov A., Capell B.C., Drake A.M., Shah P.P. (2015). Autophagy mediates degradation of nuclear lamina. Nature.

[109] Ivanov A., Pawlikowski J., Manoharan I., van Tuyn J., Nelson D.M., Rai T.S., Shah P.P., Hewitt G., Korolchuk V.I., Passos J.F. (2013). Lysosome-mediated processing of chromatin in senescence. J. Cell Biol..

[110] Lenain C., Gusyatiner O., Douma S., van den Broek B., Peeper D.S. (2015). Autophagy-mediated degradation of nuclear envelope proteins during oncogene-induced senescence. Carcinogenesis.

[111] Criscione S.W., De Cecco M., Siranosian B., Zhang Y., Kreiling J.A., Sedivy J.M., Neretti N. (2016). Reorganization of chromosome architecture in replicative cellular senescence. Sci. Adv..

[112] Bruce J.L., Hurford R.K., Classon M., Koh J., Dyson N. (2000). Requirements for cell cycle arrest by p16INK4a. Mol. Cell.

[113] Baus F., Gire V., Fisher D., Piette J., Dulic V. (2003). Permanent cell cycle exit in G2 phase after DNA damage in normal human fibroblasts. EMBO J..

[114] Gire V., Dulic V. (2015). Senescence from G2 arrest, revisited. Cell Cycle.

[115] Mattiucci D., Maurizi G., Leoni P., Poloni A. (2018). Aging- and Senescence-associated Changes of Mesenchymal Stromal Cells in Myelodysplastic Syndromes. Cell Transplant..

