# Supplementary material for: Mass Cytometry as a Tool for Investigating Senescence in Multiple Model Systems
Source: Cells. 2023 Aug 11;12(16):2045. doi: 10.3390/cells12162045 (PMC10453346; doi:10.3390/cells12162045)
Supplement: Supplementary file 1 [file cells-12-02045-s001.zip › cells-2430542-supplementary.pdf]

Figure S1

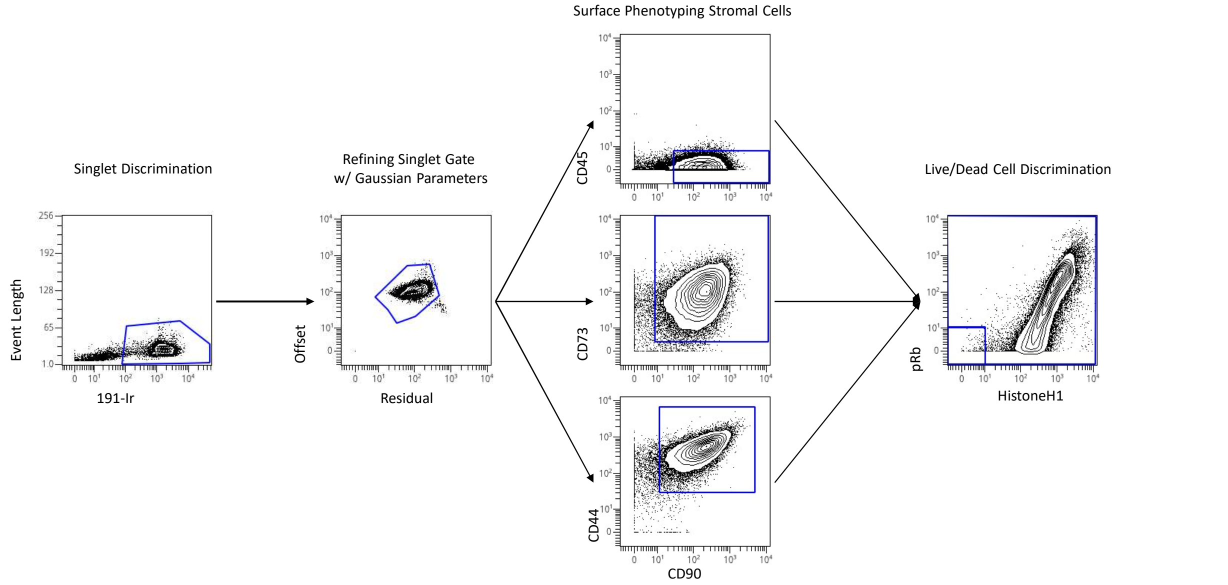

Figure S1. In order to assure only stromal cells were considered for mass cytometry (MC) analysis, a combination of surface and intracellular markers were used to isolate living stromal cells. Initial debris and doublets were removed through the use of the singlet gate, this was further refined by using the Gaussian parameters to remove additional debris and doublets. Surface markers were used next to isolate stromal cells specifically as there are multiple cell types present in femur head extractions. Once stromal cells were isolated in order to ensure only living cells were considered for senescence marker analysis the pRb by H1 gate was used to isolate living cells according to previously published studies.

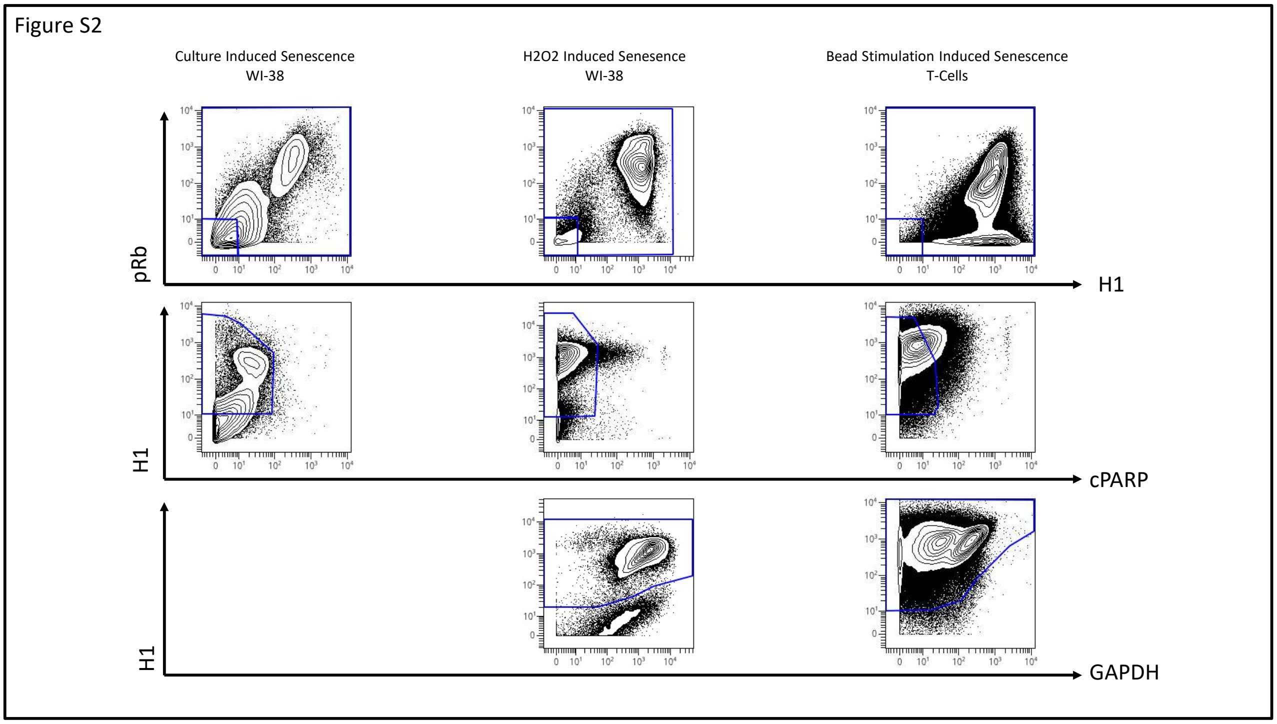

Figure S2. Gating of live cells as described previously are shown with an example of each experiment. Initial BMSC experiments did not contain the GAPDH antibody so it is excluded. Subsequent experiments included a GAPDH antibody as an additional measure of live/dead discrimination. All subsequent analysis was run on these living cells to ensure cells that were dead or dying would be excluded from the final findings.

Figure S3

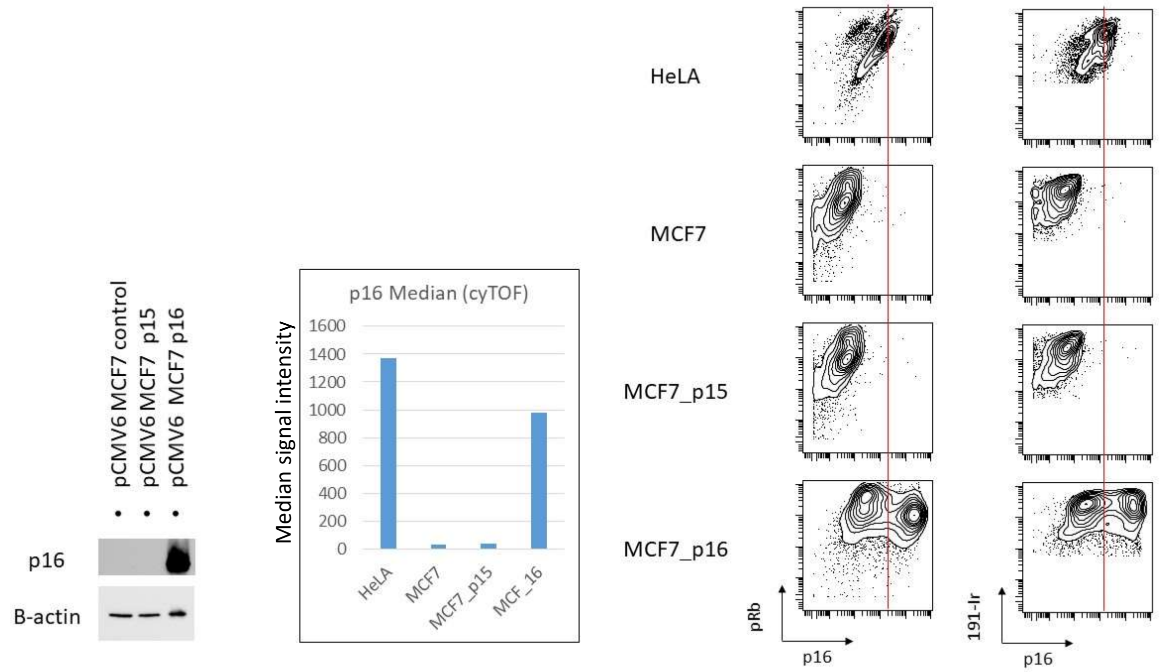

Figure S3. Validation of a p16 antibody was done in MCF7 cells that had been transfected with either a p15 or p16 construct. The expression of p16 was then validated both by western blot and mass cytometry against the standard of HeLA cells. The p15 transfection showed no cross reactivity with the p16 antibody in either western blotting or mass cytometry and this antibody was used for all future experiments.

Figure S4

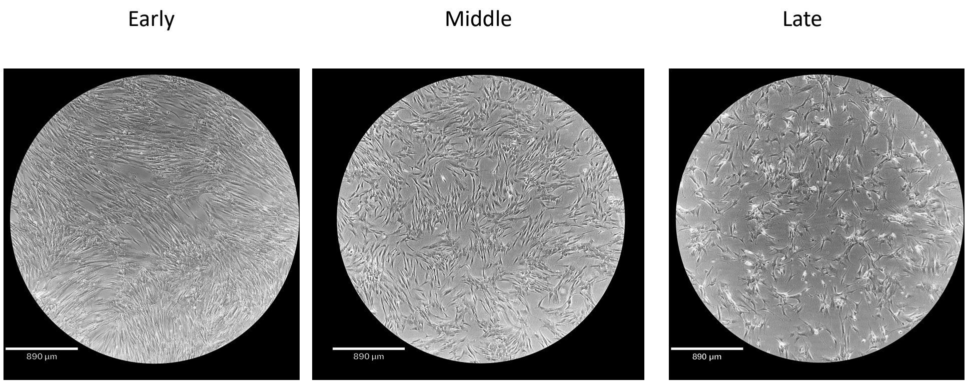

Figure S4. Representative brightfield images of one of the BMSC samples showing the morphological changes that occur during sequential passaging the development into senescence. Scale located in the lower left of each image.

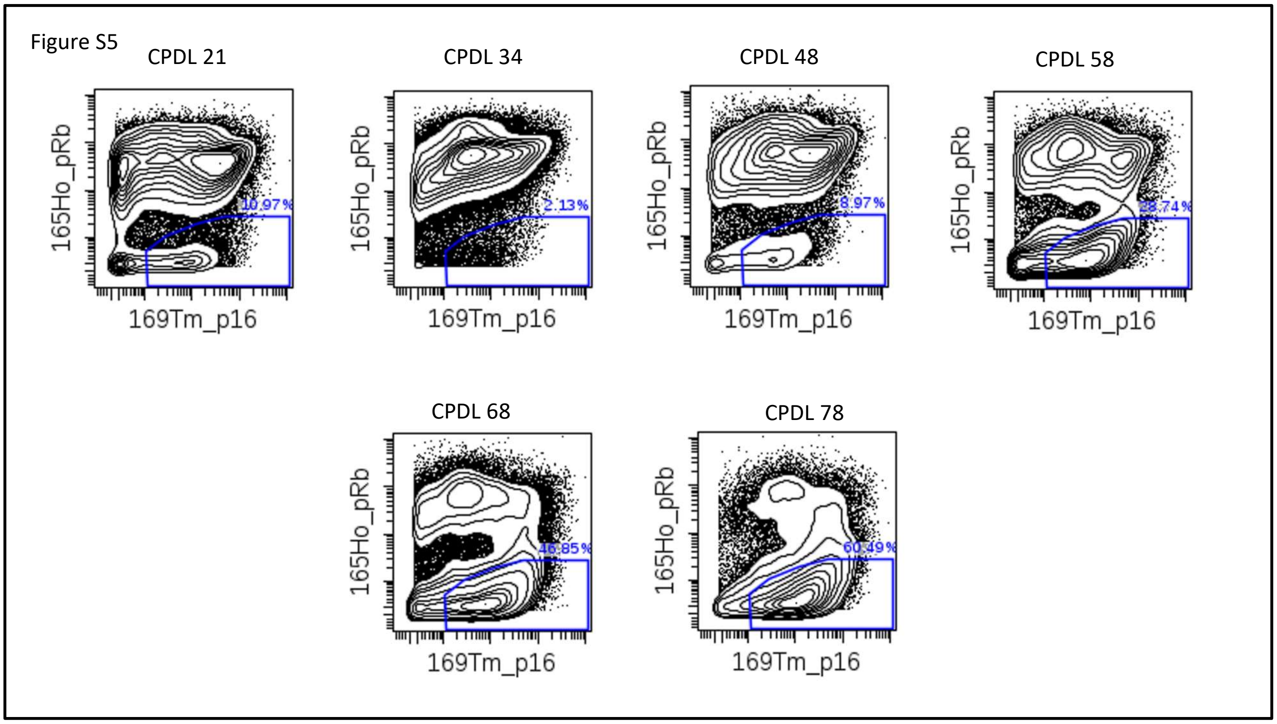

Figure S5. Changes in the p16+/pRb- expression in repeatedly cultured WI38 cells

Figure S6

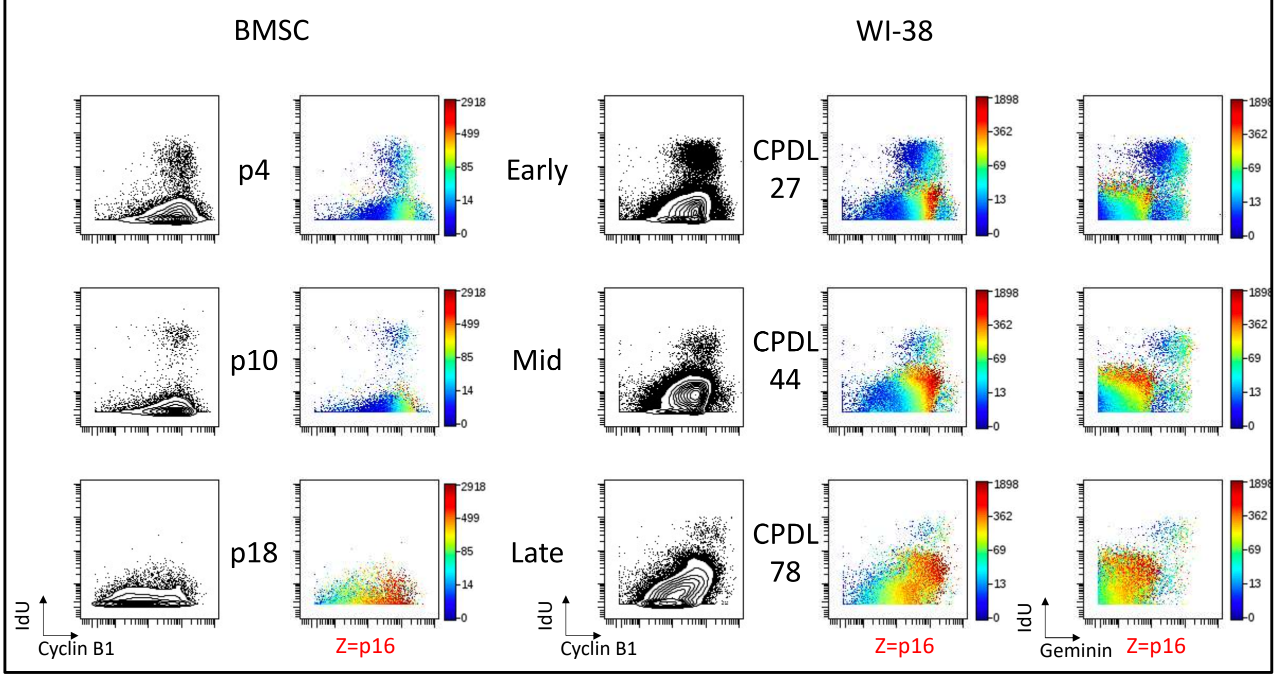

Figure S6. Expression of p16 is highest at G1-S phase boundary and at moderate levels in G0 and G1 cells at later passages. Plots of IdU (DNA synthesis) and Cyclin B1 are shown for representative BMSC (sample 151696) and WI-38 samples. Cell events are plotted as contour plots and dot plots colored for p16 expression level for early, mid, and late passages (as indicated by passage or CPDL number.

Figure S7

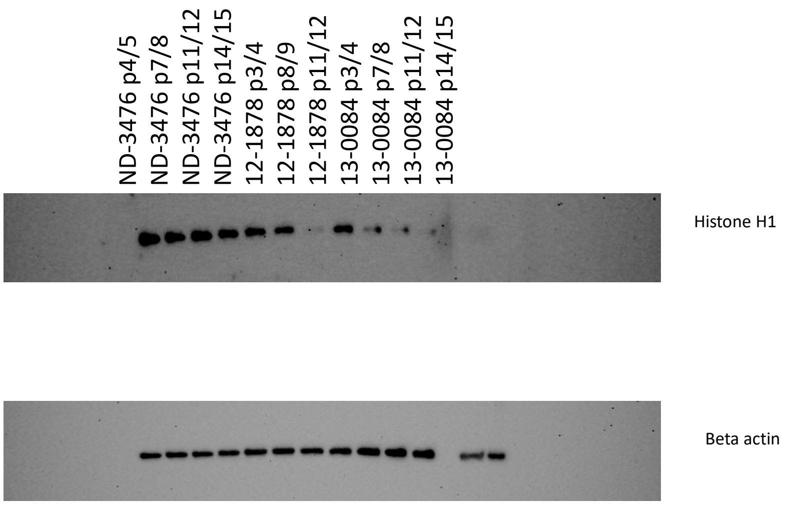

Figure S7. Stromal cells were tested for HistoneH1 expression in order to understand chromatin accessibility. In some stromal cells there was a reduced expression of HistoneH1 which has been reported previously in other studies. In some cells, however, there was no change in the expression of HistoneH1 which has also been reported in previous studies. Reduced expression of HistoneH1 is not consistent across multiple senescent models as noted in previous studies. Three primary stromal samples were tested in this western blot (alphanumerical IDs: ND-3476, 12-1878 and 13-0084).

Figure S8

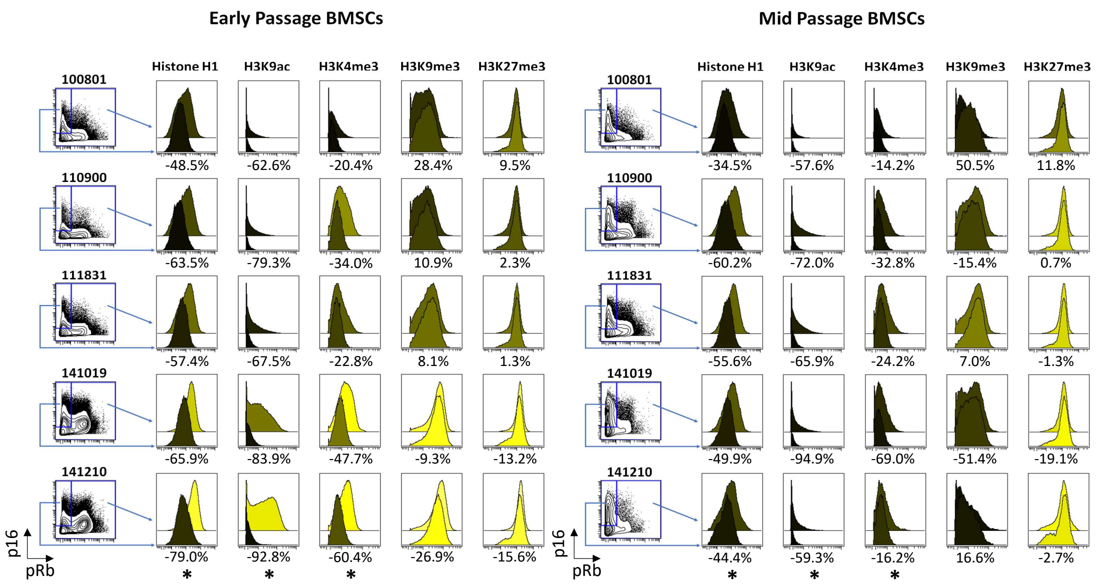

Figure S8. Changes in histone mark modifications between senescent (p16hi,pRblow; bottom histograms) vs. all other cells (top histograms) in BMSC samples from early and mid passages. Comparisons were are not shown for the late passage as many samples lacked significant numbers of non-senescent cells at this timepoint. Changes were significant (indicated by *) for total Histone H1 ($p=0.001$), H3K9ac ($p=0.037$), and H3K4me3 ($p=0.015$).

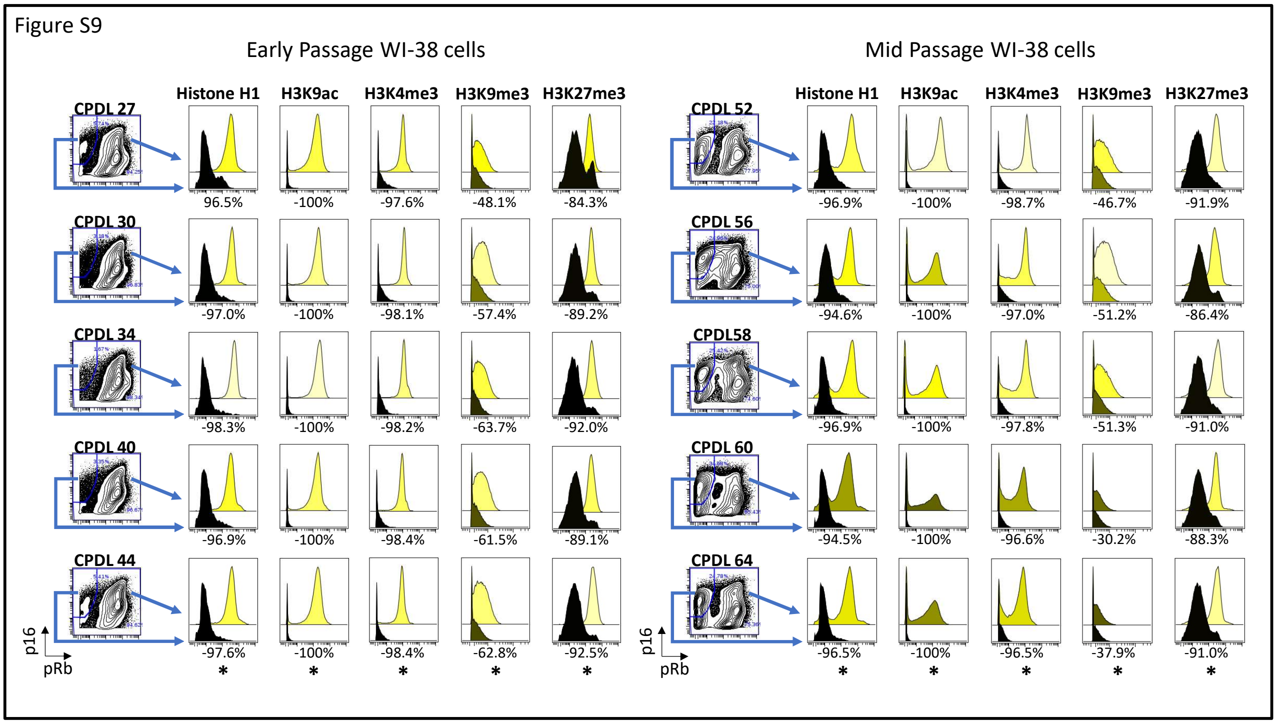

Figure S9. Changes in histone mark modifications between senescent (p16hi,pRblow; bottom histograms) vs. all other cells (top histograms) in WI38 cells from early and mid passages. Comparisons were are not shown for the late passage as many samples lacked significant numbers of non-senescent cells at this timepoint. Changes were significant (indicated by *) for all Histone measurements with a p<0.0001 (paired t-test) for all.

Figure S10

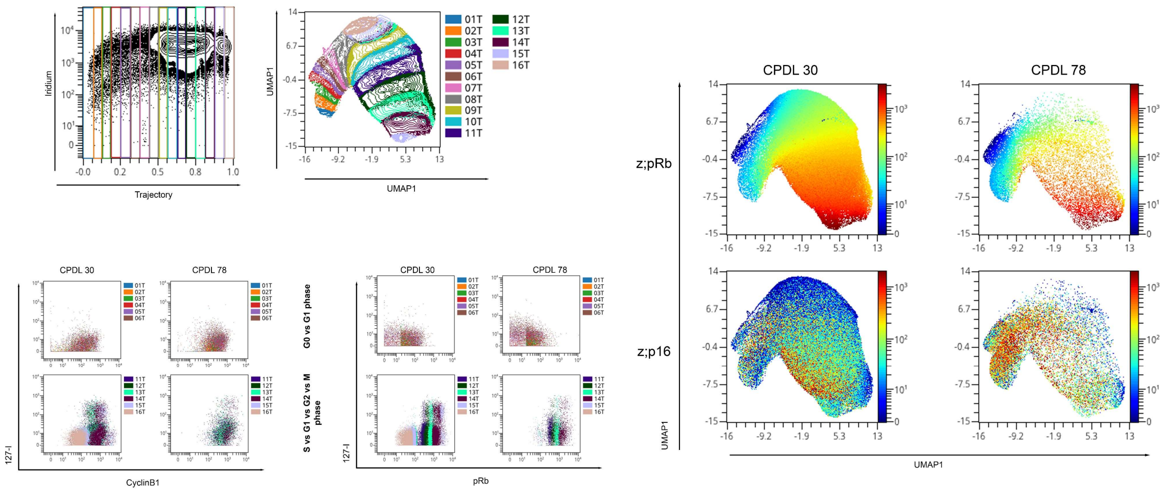

Figure S10. The wanderlust progression analysis takes the input data and slice it to develop a trajectory as shown here. The algorithm developes a trajectory parameter based on user input for the start point (0.0). This demonstrates how the trajectory parameter can be superimposed on the UMAP analysis to demonstrate the progression from the user defined start point to the end of the data. This is projected in the progression analysis as a pseudo-time across multiple samples where protein expression is plotted across the trajectory slices. In order to be consistent across multiple different model systems all progression analyses were run with the starting point set to the highest p16 expressing cells.

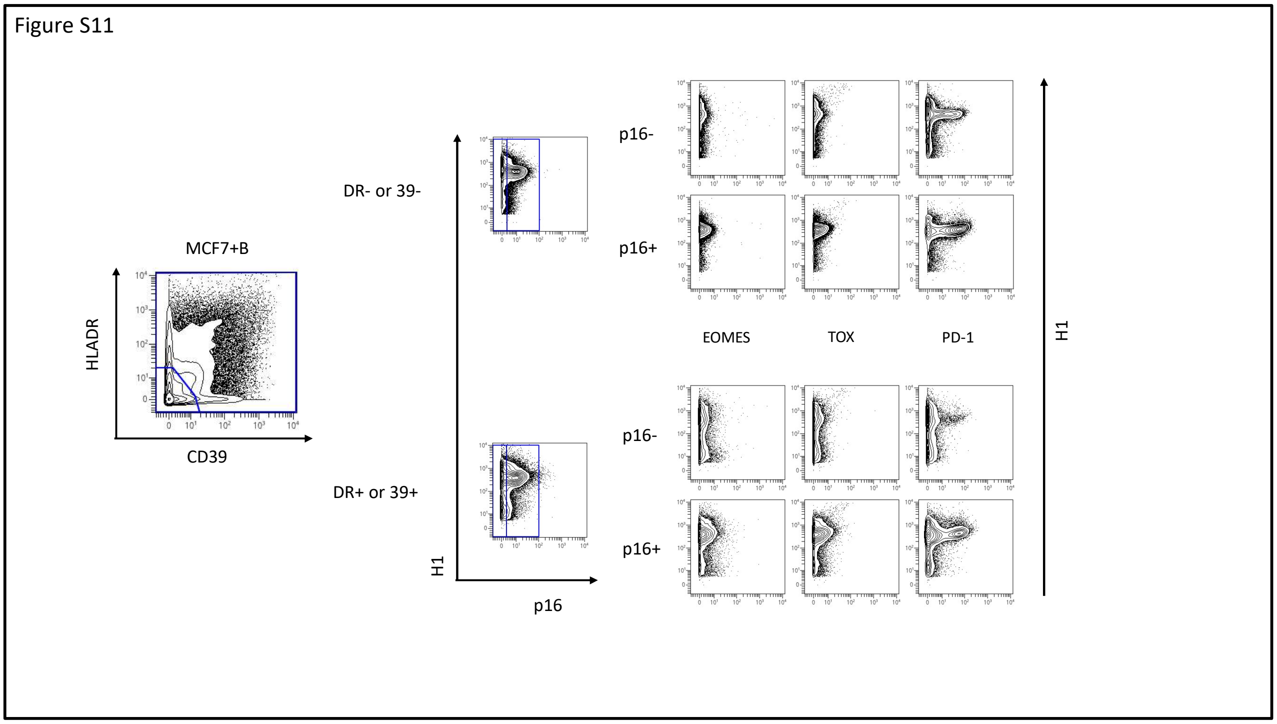

Figure S11. The co-culture and stimulation of T cells with MCF7 cells then MCF7 conditioned media demonstrated a lack of activation following antigen stimulation. This lack of activation was still associated with an increase in p16 expression along with EOMES and TOX expression. This is indicative of a suppression of activation through senescence as opposed to senescence as a consequence of repetitive stimulation

Supplementary Table S1

Antibody-Mass	Antibody	Mass	Clone #	Manufacturer
CyclinB1-156	CyclinB1	156	V152	Biolegend
pRB-165	pRB	165	J112-906	BD
pHH3-209	pHH3	209	HTA28	BioRad
Ki67-158	Ki67	158	solA15	eBioscience
p21-166	p21	166	SXM30	BD
p16-169	p16	169	D7C1M	CST
CD26-168	CD26	168	BA5b	Biolegend
H3K4me3-112	H3K4me3	112	42D8	CST
H3K27me3-194	H3K27me3	194	C36B11	CST
H1-198	H1	198	AE-4	BioRad
H3k9ac-116	H3k9ac	116	C5B11	CST
H3K79me2-141	H3K79me2	141	"15E8"	CST
H4K20me3-176	H4K20me3	176	04-079	Millipore
H2AK119Ub-159	H2AK119Ub	159	D27C4	CST
EOMES-163	EOMES	163	Dan11mag	Invitrogen
TOX-164	TOX	164	6E6D03	Biolegend
PD1-175	PD1	175	EH12.2H7	Biolegend

Supplementary Table S2

UMAP Settings	
Neighbors	15
Minimum Distance	0.4
Components	2
Metric	Euclidean
Learning Rate	1
Epoch	200

Opt-SNE Settings	
Max Iterations	1000
Opt-SNE End	5000
Perplexity	30
Theta	0.5
Components	2
Verbosity	25

Wanderlust Settings	
Waypoints	250
K	15
Filter	p16+/pRb- population

Clustering Channels
CD3
CD15
CD45
CD4
CD8
GranzymeB
HLADR
CD39
